# Synthesis and Structural Characterization of Copper Complexes Containing “R-Substituted” Bis-7-Azaindolyl Borate Ligands

**DOI:** 10.3390/molecules28124825

**Published:** 2023-06-17

**Authors:** Miriam Jackson, Simon D. Thomas, Graham J. Tizzard, Simon J. Coles, Gareth R. Owen

**Affiliations:** 1 Chemical and Environmental Sciences, Faculty of Computing, Engineering and Science, University of South Wales, Pontypridd CF37 4AT, UK; 2 UK National Crystallography Service, School of Chemistry, University of Southampton, Highfield, Southampton SO17 1BJ, UK

**Keywords:** scorpionate, copper, borohydride, ligand, nitrogen, coordination

## Abstract

The coordination chemistry of scorpionate ligands based on borates containing the 7-azaindole heterocycle is relatively unexplored. Thus, there is a requirement to further understand their coordination chemistry. This article outlines the synthesis and characterization of a family of complexes containing anionic flexible scorpionate ligands of the type [(R)(bis-7-azaindolyl)borohydride]^−^ ([**^R^Bai**]^−^), where R = Me, Ph or naphthyl. The three ligands were coordinated to a series of copper(I) complexes containing a phosphine co-ligand to form the complexes, [Cu(**^Me^Bai**)(PPh_3_)] (**1**), [Cu(**^Ph^Bai**)(PPh_3_)] (**2**), [Cu(**^Naphth^Bai**)(PPh_3_)] (**3**), [Cu(**^Me^Bai**)(PCy_3_)] (**4**), [Cu(**^Ph^Bai**)(PCy_3_)] (**5**) and [Cu(**^Naphth^Bai**)(PCy_3_)] (**6**). Additional copper(II) complexes, namely, [Cu(**^Me^Bai**)_2_] (**7**) and [Cu(**^Ph^Bai**)_2_] (**8**), were obtained during attempts to obtain single crystals from complexes **4** and **2**, respectively. Complexes **7** and **8** were also prepared independently from CuCl_2_ and two equivalents of the corresponding Li[**^R^Bai**] salt alongside an additional complex, namely, [Cu(**^Naphth^Bai**)_2_] (**9**). The copper(I) and copper(II) complexes were characterized using spectroscopic and analytical methods. Furthermore, a crystal structure was obtained for eight of the nine complexes. In all cases, the boron-based ligand was found to bind to the metal centers via a κ^3^-*N*,*N*,*H* coordination mode.

## 1. Introduction

**Figure 1:**
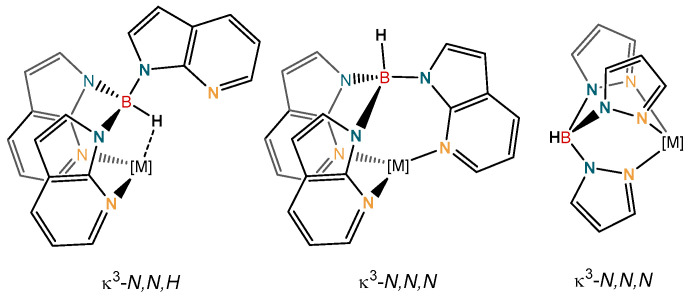
The κ^3^-*N*,*N*,*H* coordination of tris(7-azaindolyl)borate to a metal center, highlighting two six-membered rings and one eight-membered ring formed upon chelation (**left**); the κ^3^-*N*,*N*,*N* coordination of tris(7-azaindolyl)borate to a metal center, highlighting three eight-membered rings formed upon chelation (**middle**); and the κ^3^-*N*,*N*,*N* coordination of trispyrazolylborate to a metal centre, highlighting the three six-membered rings formed upon chelation (**right**).

Research involving “scorpionate ligands” has been in substantial development since the late 1960s [1,2,3,4,5]. The original Trofimenko-type scorpionate ligands were based around polypyrazolylborate ligands; however, a plethora of derivative ligands have been developed over the years. The polypyrazolylborates generally bind to metal centers via the available nitrogen donors and sometimes the B–H units, where a range of coordination modes, such as κ^3^-*N*,*N*,*N*, κ^3^-*N*,*N*,*H* and κ^2^-*N*,*N*, are common. Over the years, polypyrazolylborates gained a reputation as being robust ligands that form polycyclic chelates (dependent on the specific coordination mode). For example, the κ^3^-*N*,*N*,*N* coordination mode, which is found in the vast majority of systems, results in three six-membered rings within the resulting complex (Figure 1, right). Traditionally, they are considered inert spectator ligands that do not typically become involved within transformations at the metal center. This perspective changed following the emergence of a new set of ligand systems where the pyrazolyl heterocycle was exchanged with a heterocycle that contains an additional atom between the site of attachment to the central boron atom and the donor atom that binds to the metal center. This led to a considerable change in the landscape of the research field involving scorpionate ligands. In 2008, we suggested that this new set of ligands be termed “flexible scorpionates” [6,7] since the consequences of this extra atom in each of the heterocycles change their properties and potential reactivity quite significantly [8,9,10]. A range of heterocycles has now been utilized to generate flexible scorpionate ligands based on sulfur [9,10,11,12,13,14,15,16], nitrogen [17,18,19,20,21], phosphine [22,23], oxygen [24,25] and other donors [26,27]. To the best of our knowledge, there are only a small number of complexes reported containing nitrogen-based flexible scorpionate ligands. These are primarily based on the 7-azaindole heterocycle, as outlined herein. The tris(7-azaindolyl)borate (**Tai**) was the first to be reported by Wang in 2005 (Figure 1) [17]. This ligand was found to bind to metal centers with a range of coordination modes, including κ^3^-*N*,*N*,*N* [17], κ^3^-*N*,*N*,*H* [6,17,28,29,30,31,32] and κ^2^-*N*,*H* [33]. With the **Tai** ligand, by far the most common coordination mode was found to be κ^3^-*N*,*N*,*H* rather than κ^3^-*N*,*N*,*N* (Figure 1, left and middle). This is the opposite scenario from the Trofimenko-type trispyrazolylborates, where κ^3^-*N*,*N*,*N* is more common (Figure 1, right). The reason for this is down to the ring sizes formed upon chelation. For **Tai**, the κ^3^-*N*,*N*,*H* coordination mode provides two six-membered rings and one eight-membered ring rather than the formation of three eight-membered rings in the κ^3^-*N*,*N*,*N* mode.

**Figure 2:**
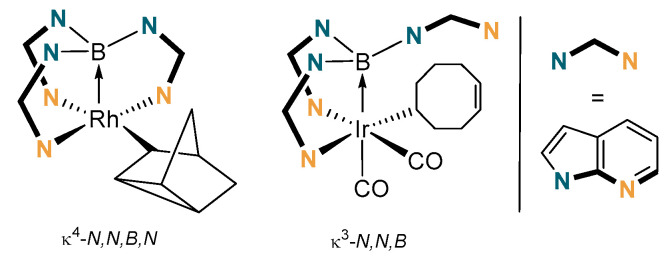
The κ^4^-*N*,*N*,*B*,*N* and κ^3^-*N*,*N*,*B* coordination modes formed by the removal of the hydride from the **Tai** ligand. In these examples, the hydride becomes incorporated into the organic co-ligand.

**Figure 3:**
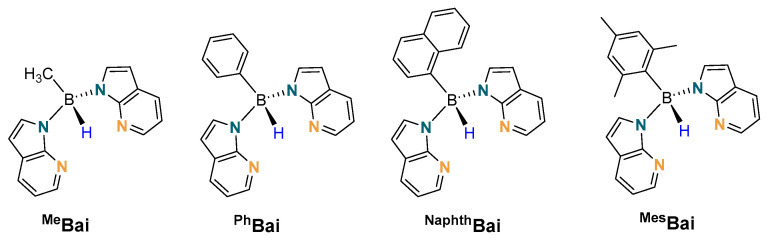
Derivative ligands, **^Me^Bai**, **^Ph^Bai**, **^Naphth^Bai** and **^Mes^Bai**.

Building on the early investigations on the **Tai** ligand [17,33], our research group has carried out subsequent research highlighting some interesting transformations. For example, we were able to demonstrate reactivity involving the borohydride unit, revealing that the **Tai** ligand is able to form metallaboratrane-type complexes [34], where the hydride is removed from the ligand and the resulting borane ligand binds with either κ^4^-*N*,*N*,*B*,*N* or κ^3^-*N*,*N*,*B* coordination modes (Figure 2) [29,31,35,36]. We additionally prepared several complexes containing straightforward coordination of the **Tai** ligand [6,28,29,30,31,32]. Furthermore, we also prepared four derivative ligands in which one of the 7-azaindolyl substituents was replaced by an alkyl or aryl substituent and further investigated their coordination properties (Figure 3) [29,37,38]. The four ligands, namely, **^Me^Bai**, **^Ph^Bai**, **^Naphth^Bai** and **^Mes^Bai**, contain a central borohydride unit containing two 7-azaindolyl substituents and one additional R group (i.e., either methyl, phenyl, naphthyl or mesityl). In all cases, these ligands adopt the archetypical κ^3^-*N*,*N*,*H* coordination mode (unless there is an activation of the B–H bond), where the R group points away from the transition metal center and the B–H---M interactions appear to be strong [28,32,38]. Despite this, even though the R group is positioned away from the metal centers upon coordination, it was found that the group influences the properties and subsequent reactivity of the resulting complexes quite significantly. For example, in a family of complexes of the type [RuH({**^R^Bai**}(PPh_3_)_2_] containing ligands with aromatic R groups, it was found that the naphthyl ring in **^Naphth^Bai** demonstrated hindered rotation about the B–C bond of the ligand [30]. Across several examples, we observed different product selectivities depending on the identity of the third “arm” of the ligand [29,36,37]. With limited knowledge about these ligands, there is a requirement to further explore the coordination properties of these derivative ligands. Therefore, we initiated an investigation to prepare a range of copper(I) phosphine and copper(II) complexes containing the **^Me^Bai**, **^Ph^Bai** and **^Naphth^Bai** ligands. This knowledge, particularly a deeper understanding of the nature of the interaction of the BH unit with the metal centers, will be invaluable in tuning the potential reactivity and exploring future applications. Herein, we outline the synthesis, characterization and structural characterization of a series of nine copper complexes containing these ligands.

## 2. Results and Discussion

### 2.1. Synthesis and Characterisation of Copper(I) Phosphine Complexes

**Scheme 1:**
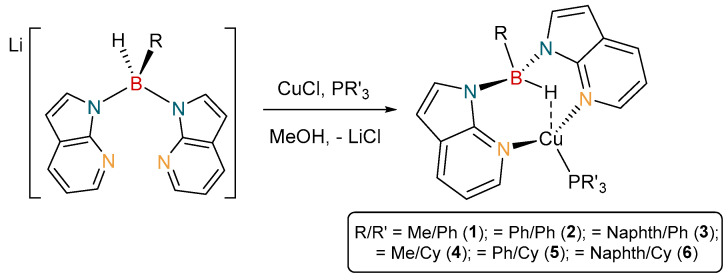
Synthesis of [Cu{κ^3^-*N*,*N*,*H*-**^R^Bai**}(PR_3_)] complexes **1**–**6**.

The synthesis of copper(I) phosphine complexes containing scorpionate ligands is well known. They are readily synthesized via standard literature protocols [14,39]. Despite this, there is only one example of a copper complex containing a 7-azaindolyl-borate-based ligand known [17]. Therefore a set of copper complexes with the general formula [Cu(**^R^Bai**)(PR’_3_)] (where R = Me, Ph or Naphth and R’ = Ph or Cy) were prepared. These were synthesized via a direct reaction of stoichiometric quantities of copper(I) chloride and the corresponding ligand precursor Li[**^R^Bai**] in the presence of one equivalent of either triphenylphosphine or tricyclohexylphosphine (Scheme 1). The reactions were performed in methanol solvent from which the products precipitated out as white/off-white solids. These solids were washed further with acetonitrile and subsequently dried to provide the products, namely, complexes **1**–**6**, in yields ranging between 72% and 92% (see the Materials and Experimental Methods section for details; the ^1^H, ^13^C{^1^H}, ^31^P{^1^H} and ^11^B{^1^H} NMR spectra and mass spectrometry data for these complexes are provided in the Supplementary Materials).

The solid products were found to be air and moisture stable. They demonstrated solubility in chlorinated solvents and partial solubility in aromatic solvents, acetonitrile and diethyl ether. It should be noted that they were found to decompose in tetrahydrofuran solutions. They were characterized by multinuclear NMR spectroscopy, IR spectroscopy and mass spectrometry. Selected characterization data for complexes **1**–**6** are presented in Table 1, along with data for the corresponding lithium ligand salt precursors for comparison. First, the ^31^P{^1^H} NMR spectra for complexes **1**–**6** in C_6_D_6_ revealed one single resonance with a moderate downfield shift with respect to their respective ligand precursors, namely, PPh_3_ (−5.26 ppm) and PCy_3_ (9.86 ppm). The phosphorus signals in the spectra for complexes **1**, **2** and **3** were located at 1.41 ppm, 1.17 ppm and 1.64 ppm, respectively, whilst the corresponding signals in **4**, **5** and **6** were found at 17.74 ppm, 18.08 ppm and 18.63 ppm, respectively. These values are in agreement with similar related scorpionate copper(I) phosphine complexes [14,17,39]. The ^11^B NMR spectra for complexes **1**–**6** also revealed a single broad resonance (with half-height widths ranging between 180 Hz and 213 Hz), with chemical shifts in the region between −6.23 ppm and −7.65 ppm. The ^11^B NMR signals would be expected to present as doublet signals due to the presence of the BH unit in the ligand (i.e., ^1^J_BH_ coupling). The peak widths, however, were too large to directly observe any coupling. Nevertheless, tentative evidence for the coupling was obtained in the corresponding ^11^B{^1^H} NMR spectra, where the half-height widths of the signals were reduced to between 129 Hz and 121 Hz. It should be noted that these chemical shifts were only slightly shifted (ca. 1 ppm downfield) with respect to their corresponding ligand salt precursors. In particular, the ^11^B NMR shifts for the Cu(**^Naphth^Bai**)(PR’_3_) complexes were almost the same as the shifts for Li[**^Naphth^Bai**]. These rather small downfield shifts upon coordination to copper centers are typical for copper complexes of this type, and thus, are expected [14,17,39]. Whilst this would seem to indicate that either there is no interaction or only a weak interaction between the BH units and the copper centers, the corresponding ^1^H NMR and IR spectroscopic evidence and structural data outlined below are contrary to this (vida infra).

For our initial analysis, we performed the NMR spectroscopic experiments in CDCl_3_. However, the resulting spectra, particularly the ^1^H and ^1^H{^11^B} NMR spectra, were poorly resolved. We found that the spectra were moderately improved and better resolved in C_6_D_6_. In all cases, the chemical shift patterns in both the ^1^H and ^13^C{^1^H} spectra, and integrations within the ^1^H NMR spectra, of the complexes were consistent with the presence of two 7-azaindolyl units, one phosphine ligand and one R group. This confirmed that all products were of the formula [Cu(**^R^Bai**)(PR’_3_)]. The ^1^H{^11^B} NMR spectra were utilized to obtain the chemical shift of hydrogen nuclei within the B–H---Cu unit. This is also included in Table 1. An examination of the aromatic region of the ^1^H NMR spectra for complexes **1**–**6** demonstrated that the two azaindolyl rings within the ligand were chemically equivalent on the NMR timescale. For the bulkier naphthyl unit in the **^Naphth^Bai** complexes, hindered rotation might have been present, as was observed in a previous example [30]. This was found not to be the case in the copper complexes **3** and **6**. The B*H* resonances within these complexes are also of interest. These were located in the δ 5.49–6.97 region ppm for all complexes. These appeared as extremely broad signals in the standard ^1^H NMR spectra. They did appear as broadened singlets in the corresponding ^1^H{^11^B} NMR spectra for complexes **2**, **3**, **5** and **6**. In the cases of **1** and **4**, quartet signals were expected. These signals within the spectra did show some apparent coupling; however, this was not resolved. In comparison with the ligand salt precursors, these signals were shifted downfield by approximately 1 ppm in each case upon coordination with the copper centers. Finally, the proton signals corresponding to the BCH_3_ unit were observed within the ^1^H NMR spectra for **1** and **4**. These appeared as more resolved doublets in the corresponding ^1^H{^11^B} NMR spectra at 1.15 ppm (^3^J_HH_ = 4.2 Hz) and 1.17 ppm (^3^J_HH_ = 4.4 Hz), respectively.

The B–H---Cu units in the six complexes were also explored using IR spectroscopy (see Table 1). In each case, powder film samples gave two characteristic bands between 2062 cm^−1^ and 2090 cm^−1^ for the first band and 2120 cm^−1^ and 2189 cm^−1^ for the second band. A large reduction in the stretching frequencies with respect to the lithium salt precursors confirmed a significant interaction of the B–H units with the copper centers, and thus, suggested the κ^3^-*N*,*N*,*H* coordination mode for the **^R^Bai** ligands. The compounds were also analyzed using mass spectrometry. The molecular ion peaks were found in most cases with two exceptions, where expected fragmentation had occurred under the mass spectrometry conditions. Interestingly, complex **4** exhibited luminescence under UV light in the solid state at room temperature. This is the only complex within this investigation to exhibit this behaviour.

### 2.2. Structural Characterization of Copper(I) Phosphine Complexes

**Figure 4:**
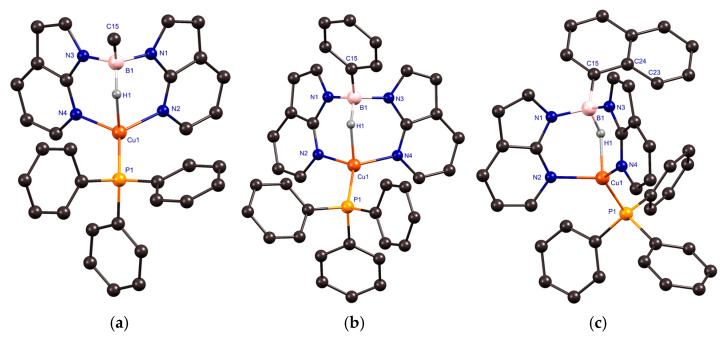
Molecular structures of [Cu{κ^3^-*N*,*N*,*H*-R(H)B(7-azaindolyl)_2_}(PPh_3_)] (R = Me, **1** (**a**); Ph, **2** (**b**); naphthyl, **3** (**c**)). Ball and stick representations. Hydrogen atoms, with the exception of those attached to the boron centers, are omitted for clarity.

Single crystals of all six complexes were obtained, allowing for a detailed comparison across the three different scorpionate ligands. The first three structures analyzed were those containing the triphenylphosphine co-ligand. Single crystals of **1** were obtained via the slow evaporation of a methanolic solution, whilst single crystals of **2** and **3** were grown via the slow evaporation of acetonitrile from their saturated solutions. Complex **1** crystallized as colorless prism-shaped crystals, **2** as colorless rod-shaped crystals and **3** as colorless needle-shaped crystals. The structure for **3** contained a molecule of acetonitrile solvent of crystallization within the asymmetric unit. The molecular structures of these complexes are shown in Figure 4. Selected bond distances and angles for these complexes are shown in Table 2. Details on data collection and crystallographic parameters for all structures are provided in the Supplementary Materials.

The structures of **1**–**3** confirmed the coordination of one triphenylphosphine ligand and one [**^R^Bai**]^−^ ligand to the copper centers. In all three complexes, the scorpionate ligand was coordinated via the two pyridyl nitrogen donors on the azaindolyl rings and the borohydride unit, thus adopting a κ^3^–*N*,*N*,*H* coordination motif. The Cu(1)∙∙∙H(1) distances for the three complexes were found to be 1.847(8) Å (for **1**), 1.813(9) Å (for **2**) and 1.803(11) Å (for **3**). These are typical for complexes of this type (c.f. the sum of the covalent radii of Cu and H = 1.63 Å [40]). The Cu---H interaction decreased on changing from Me to Ph to Naphth. The corresponding B(1)–H(1) distances for **1**–**3** were 1.230(8) Å, 1.268(9) Å and 1.282(12) Å, respectively. The Cu(1)∙∙∙H(1)–B(1) interaction impacted the overall geometry of the copper centers. The geometry at the metal centers was somewhat distorted between tetrahedral and trigonal pyramidal. The sums of the angles defining the trigonal plane, involving a copper atom, two nitrogen atoms and phosphine, were 356.89(3)° for **1**, 349.07(3)° for **2** and 350.49(9)° for **3**. The idealized bond angles for trigonal pyramidal are 120° and 90°, whilst for trigonal pyramidal, it is 109.5°. Ignoring those involving the hydrogen atom (which would sit on the axial site of the former geometry), complexes **1**, **2** and **3** had angles ranging between 107.73(2) and 125.192(16), 108.70(2) and 121.888(19) and 107.27(3) and 124.43(3), respectively. The N(2)–Cu–N(4) angles that arose from the chelation of the **^R^Bai** ligand provided the smallest of the aforementioned angles. The only other known copper complex containing a 7-azaindolyl-based scorpionate ligand is [Cu(**Tai**)(PPh_3_)] [17]. This adopts the same coordination mode (i.e., κ^3^-*N*,*N*,*H*) as found in the copper complexes reported herein despite the fact that the **Tai** ligand contains three 7-azaindolyl “arms”. In [Cu(**Tai**)(PPh_3_)], the pyridyl unit of the uncoordinated 7-azaindolyl points directly away in the opposite direction from the Cu∙∙∙H–B vector, where the Cu--B–N---N torsion angle is 178.6(2)^°^. In the case of complexes **2** and **3**, the aryl groups were orientated at quite different positions with respect to the Cu∙∙∙H–B vector. In the case of **2**, the smallest M--B–C_ipso_–C_ortho_ torsion angle was 64.48(8)°, whilst in **3**, the corresponding angle was 57.66(14)°. These showed that the aryl groups in the **^Ph^Bai** and **^Naphth^Bai** complexes had twisted orientations relative to the Cu∙∙∙H–B unit, where the aryl group avoided steric clashes with the C–H bonds of the azaindolyl rings. Interestingly, the packing within the structure for **1** showed π-π stacking involving one of two azaindolyl “arms” of the ligand on one complex with the same on the adjacent complex. The interaction involved the pyridyl ring units, where the distance between the two centroids defined by the six atoms within each of the pyridyl rings was 3.62841(3) Å. There were no apparent π-π stacking interactions in the other two complexes containing the **^Ph^Bai** and **^Naphth^Bai** ligands. The reason behind this was most likely due to the smaller volume of space the methyl group occupied, allowing for the azaindolyl units to become closer to each other.

For the most part, the bonding features involving the “Cu{BH(7-azaindolyl)_2_(PPh_3_)]” core, which is common to all of the aforementioned complexes, were similar to each other. Notable exceptions were the N–B–N bond angles involving the two coordinated 7-azaindolyl units, which were 109.77(5)° (for **1**), 109.42(6)° (for **2**), 106.89(8)° (for **3**) and 111.7(3)° (for [Cu(**Tai**)(PPh_3_)]). This suggested some influence of the third “arm” of the ligand on the coordination of the ligand within the complexes.

**Figure 5:**
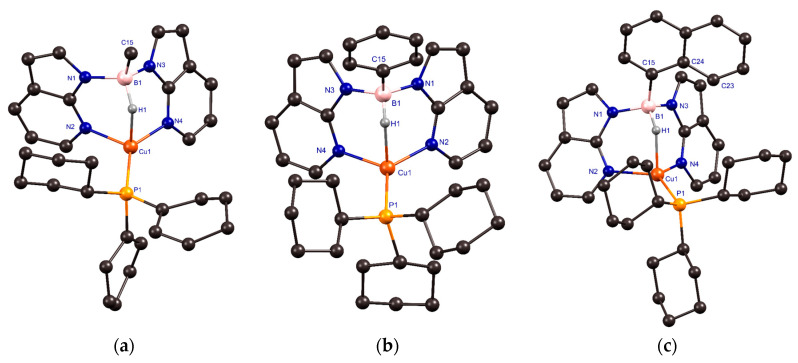
Molecular structures of [Cu{κ^3^-*N*,*N*,*H*-R(H)B(7-azaindolyl)_2_}(PCy_3_)] (R = Me, **4** (**a**); Ph, **5** (**b**); naphthyl, **6** (**c**)). Ball and stick representations. Hydrogen atoms, with the exception of the one attached to each of the boron centers, are omitted for clarity.

The crystal structures of three complexes containing the tricyclohexylphosphine co-ligand were analyzed next (Figure 5). Single crystals of [Cu(**^Me^Bai**)(PCy_3_)] (**4**) were obtained via concentration of a saturated methanol solution, whilst crystals of [Cu(**^Ph^Bai**)(PCy_3_)] (**5**) and [Cu(**^Naphth^Bai**)(PCy_3_)] (**6**) were grown via slow evaporation from saturated acetonitrile solutions. All three complexes crystallized as colorless block-shaped crystals. The calculated structure for **6** was found to contain one molecule of acetonitrile solvent within the asymmetric unit. Selected bond distances and angles for these complexes are shown in Table 3.

Complexes **4**, **5** and **6** share many similarities with the corresponding triphenylphosphine complexes discussed above. Most bonding parameters were analogous to those of complexes **1**–**3**. In particular, the crystal structures also confirmed the κ^3^–*N*,*N*,*H* coordination modes of the **^R^Bai** ligands within these complexes. As a general trend, the Cu(1)–P(1) distances in these three complexes were slightly longer than the corresponding triphenylphosphine complexes. This was due to the greater steric bulk of the tricyclohexylphosphine co-ligand. The positioning of the aryl rings with respect to the Cu∙∙∙H–B vector in **5** and **6** was larger than those found in **2** and **3**, where minor differences can be attributed to slightly increased N(1)–B(1)–N(3) angles within these PCy_3_ complexes. The smallest M--B–C_ipso_–C_ortho_ torsion angles in **5** and **6** were 87.5(4)° and 70.00(6)°. As with the **^Me^Bai** complex **1**, there was also π-π stacking in complex **4**. This again involved one of two azaindolyl “arms” on one complex with the same on the adjacent complex. In this case, however, the interaction involved the indolyl units where the distance between the two centroids defined by the five atoms within each of the indolyl rings was 3.72074(12) Å. As with **2** and **3**, there were no apparent π-π stacking interactions in the structures for **5** and **6**.

### 2.3. Synthesis and Crystallization of Copper(II) Complexes

**Scheme 2:**
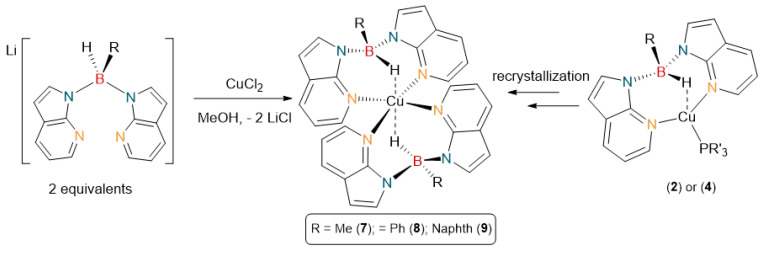
Synthesis of [Cu{κ^3^-*N*,*N*,*H*-**^R^Bai**}_2_] complexes **7**–**9**, either directly from CuCl_2_ and two equivalents of pro-ligand or obtained via recrystallized attempts from **2** and **4**.

During our investigations and attempts to obtain crystal structures for complexes **2** and **4** above, we also isolated single crystals, which were solved as [Cu(**^R^Bai**)_2_] (where R = Me (**7**) and Ph (**8**)). The analysis of their structures is outlined below. These two complexes, along with the additional complex [Cu(**^Naphth^Bai**)_2_] (**9**), were prepared independently via a reaction of copper(II) chloride with two equivalents of the corresponding Li[**^R^Bai**] ligand precursor in methanol at room temperature (Scheme 2). The three complexes were obtained in high yields between 75 and 91%. The IR spectra for the three paramagnetic compounds each showed single bands at 2226 cm^−1^ (for **7**), 2269 cm^−1^ (for **8**) and 2221 cm^−1^ (for **9**). These corresponded to the coordinated B–H stretching frequency, confirming the κ^3^-*N*,*N*,*H* coordination mode of the two ligands in each complex. In these cases, the reduction of the stretching frequencies within these bis-ligand complexes compared with the ligand precursors was less pronounced, suggesting that the Cu∙∙∙H–B interactions were weaker. This may be related to the increased coordination number of the copper centers in **7**–**9** in comparison with the tetracoordinated copper centers in complexes **1**–**6**. Mass spectrometry (ES^+^) confirmed the molecular composition of the complexes with the following molecular ion peaks: 586 a.m.u. for [Cu(**^Me^Bai**)_2_]^+^, 710 a.m.u. for [Cu(**^Ph^Bai**)_2_]^+^ and 809 a.m.u. for [Cu(**^Naphth^Bai**)_2_]^+^.

**Figure 6:**
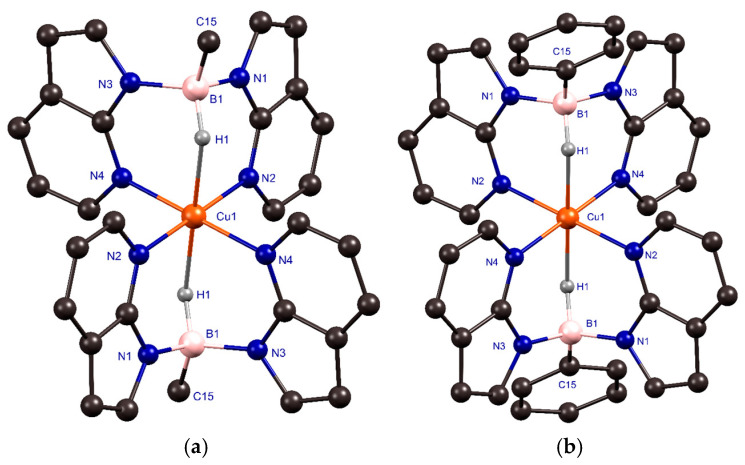
Molecular structures of [Cu{κ^3^-*N*,*N*,*H*-R(H)B(7-azaindolyl)_2_}_2_] (R = Me, **7** (**a**); Ph, **8** (**b**)). Ball and stick representations. Hydrogen atoms, with the exception of those attached to the boron centers, are omitted for clarity.

The formation of the mononuclear bis-ligand complexes [Cu(**^Me^Bai**)_2_] (**7**) and [Cu(**^Ph^Bai**)_2_] (**8**) was confirmed via X-ray crystallography (Figure 6). Selected bond distances and angles for these two complexes are shown in Table 4. Single crystals of **7** were obtained via concentration of a methanol solution containing complex **4**. Crystals of **8** were obtained by layering a concentrated dichloromethane solution of complex **2** with hexane. Complex **7** crystallized as brown prisms, containing one complex within the asymmetric unit, whilst complex **8** crystallized as light brown blocks, with two independent complexes and a disordered molecule of dichloromethane solvent within the unit cell. In the cases of both **7** and **8**, the asymmetric unit consisted of half of each complex (i.e., [Cu(**^R^Bai**)].

The crystal structures confirmed the coordination of two **^R^Bai** ligands, both with κ^3^-*N*,*N*,*H* coordination modes, as expected. This resulted in octahedral copper(II) centers, where the BH units were positioned *trans* to each other and all four pyridyl nitrogen donors of the two scorpionate ligands lay on a crystallographic plane. The N(2)–Cu–N(4) angles that arose from the chelation of the **^R^Bai** ligands were significantly smaller in these complexes compared with the copper(I) phosphine complexes outlined above. This was to accommodate the change in geometry at the copper centers. Interestingly, the intra-ligand N–Cu–N angle in **7** was smaller than the inter-ligand N–Cu–N angle, c.f. 89.77(6)° with 90.23(6)°. The opposite was found in the case of **8**, which had angles of 92.94(5)° and 91.10(5)° for the intra-ligand angles and 87.06(5)° and 88.90(5)° for the inter-ligand angles. There appeared to be great flexibility in the κ^3^-*N*,*N*,*H* coordination and chelation of the **^R^Bai** ligands. The N–Cu–N angle may have had an impact on the positioning of the BH units within the complexes, reducing their interaction with the copper centers. This is of interest since the copper centers in **7** and **8** are in a higher oxidation state [c.f. Cu(II) vs. Cu(I)] and higher coordination number (c.f. six-coordinate vs. four-coordinate). The Cu(1)---B(1) distances in complexes **1** – **6** above ranged between 2.763(2) Å and 2.7995(7) Å. The boron centers were further away in the bis-ligand complexes. In these cases, the Cu(1)---B(1) distances ranged between 2.9281(15) Å and 2.944(2) Å. The corresponding Cu(1)∙∙∙H(1) distances in **7** and **8** ranged between 2.00(2) Å and 2.08(2) Å. Finally, as with the two structures containing the **^Me^Bai** above, there was π-π stacking in complex **7**. This involved one pyridyl component within one complex with an indolyl component in an adjacent complex. The distance between the two respective centroids was 3.82028(11) Å. The π-π stacking appeared to be specifically related to the steric properties of this ligand in particular.

## 3. Materials and Experimental Methods

The syntheses of the complexes were carried out using standard Schlenk techniques. Solvents were sourced as extra dry from “*Acros Organics*” and were stored over either 4 Å or 3 Å molecular sieves. The NMR solvents, CDCl_3_ and C_6_D_6_ were stored in a Young’s ampule over 4 Å molecular sieves under a N_2_ atmosphere and were degassed through freeze−thaw cycles prior to use. Reagents were used as purchased from commercial sources. The ligand salts Li[**^R^Bai**] (where R = Me, Ph or Naphth) [29,37,38] were synthesized according to standard literature procedures. NMR spectroscopy experiments were conducted on a Bruker 400 MHz AscendTM 400 spectrometer. All spectra were referenced internally to the residual protic solvent (^1^H) or the signals of the solvent (^13^C). Proton (^1^H) and carbon (^13^C) assignments were further supported using HSQC, HMBC and COSY NMR experiments. In these cases, the apparent coupling constant is provided. Infrared spectra were recorded on a PerkinElmer Spectrum Two ATR FT-IR spectrometer as powder films. Elemental analysis was performed at London Metropolitan University using their elemental analysis service. Mass spectra were recorded at Cardiff University analytical services. Crystallographic details on data collection and parameters are outlined in the Supplementary Materials.

### 3.1. Synthesis of [Cu{κ^3^-N,N,H-**^Me^Bai**}(PPh_3_)] (**1**)

A clean dry Schlenk flask was charged with CuCl (50 mg, 0.51 mmol), ligand precursor [Li(**^Me^Bai**)] (133 mg, 0.51 mmol) and PPh_3_ (262 mg, 1 mmol). Methanol (20 mL) was subsequently added, and the reaction mixture was stirred for 2 h at room temperature. The solvent was then removed via filtration to isolate the precipitate [Cu(**^Me^Bai**)(PPh_3_)] as a white solid, which was washed with acetonitrile and dried (247 mg, 0.42 mmol, 83%). NMR δ ppm: ^1^H (C_6_D_6_, 400 MHz), 7.94 (d, ^3^J_HH_ = 5.02 Hz, 2H, ^Aza^CH), 7.85 (d, ^3^J_HH_ = 3.2 Hz, 2H, ^Aza^CH), 7.53–7.59 (overlapping m, 8H, P(C_6_H_5_) + ^Aza^CH), 6.92–7.00 (m, 9H, P(C_6_H_5_)), 6.51 (dd, ^3^J_HH_ = 5.95 Hz, 2H, ^Aza^CH), 6.43 (d, ^3^J_HH_ = 3.24 Hz, 2H, ^Aza^CH), 5.63 (d, ^3^J_HH_ = 2.74 Hz, H, BH), 1.15 (broad doublet, 3H, BCH_3_, apparent J_HH_ = 3.77 Hz, this signal became more resolved in the corresponding ^1^H{^11^B} experiment, ^3^J_HH_ = 4.24 Hz), ^13^C{^1^H} (C_6_D_6_, 100 MHz), 151.3 (^Aza^C), 140.8 (^Aza^CH), 134.4 (d, ^1^J_CP_ = 31.1 Hz, P^ipso^(C_6_H_5_)), 134.3 (d, ^2^J_CP_ = 15.4 Hz, P^ortho^(C_6_H_5_)), 132.1 (^Aza^CH), 130.2 (d, ^4^J_CP_ = 1.4 Hz, P^para^(C_6_H_5_)), 129.2 (d, ^3^J_CP_ = 9.5 Hz, P^meta^(C_6_H_5_)), 128.9 (^Aza^CH), 124.8 (^Aza^C), 114.0 (^Aza^CH), 100.6 (^Aza^CH), 1.5 (BCH_3_). ^31^P{^1^H} NMR (δ, C_6_D_6_): 1.41. ^11^B NMR (δ, C_6_D_6_): −7.27 (s, h.h.w. = 186 Hz). ^11^B{^1^H} NMR (δ, C_6_D_6_): −7.27 (s, h.h.w. = 129 Hz). IR (cm^−1^, powder film): 2089, 2143. MS ES+ (*m*/*z*): 587 [Cu(**^Me^Bai**)PPh_3_]^+^.

### 3.2. Synthesis of [Cu{κ^3^-N,N,H-**^Ph^Bai**}(PPh_3_)] (**2**)

A clean dry Schlenk flask was charged with CuCl (100 mg, 1.00 mmol), ligand precursor [**Li**(**^Ph^Bai**)**]** (330 mg, 1.00 mmol) and PPh_3_ (262 mg, 1.00 mmol). Methanol (20 mL) was subsequently added and the reaction mixture was stirred for 2 h at room temperature. The solvent was then removed via filtration and the precipitate [Cu(**^Ph^Bai**)(PPh_3_)] was washed with acetonitrile (2 × 10 mL) to give the product as a white solid (598 mg, 0.92 mmol, 92%). NMR δ ppm: ^1^H (C_6_D_6_, 400 MHz), 7.93 (d, ^3^J_HH_ = 4.81 Hz, 2H, ^Aza^CH), 7.84 (3H, overlapping ^Aza^CH + (C_6_H_5_)), 7.52 (m, 8H, P(C_6_H_5_) + (C_6_H_5_)), 7.38 (t, ^3^J_HH_ = 7.52 Hz, 2H, ^Aza^CH), 7.30 (t, ^3^J_HH_ = 7.89 Hz, 2H, (C_6_H_5_)), 6.88–6.96 (m, 9H, P(C_6_H_5_)), 6.58 (unresolved, H, BH), 6.53 (dd, ^3^J_HH_ = 2.5 Hz, ^3^J_HH_ = 5.17 Hz, 2H, ^Aza^CH), 6.42 (d, ^3^J_HH_ = 3.27 Hz, 2H, ^Aza^CH). ^13^C {^1^H} (C_6_D_6_, 100 MHz), 151.6 (^Aza^C), 140.3 (^Aza^CH), 134.0 (^Aza^CH), 133.7 (d, ^2^J_CP_ = 15.4 Hz, P^ortho^(C_6_H_5_)), 133.14 (^Aza^CH), 129.6 (d, ^4^J_CP_ = 1.2 Hz, P^para^(C_6_H_5_)), 128.6 (d, ^3^J_CP_ = 10.0 Hz, P^meta^(C_6_H_5_)), 127.7 (d (overlapped with C_6_D_6_), ^1^J_CP_ = 23.9 Hz, P^ipso^(C_6_H_5_)), 127.0 (C_6_H_5_), 125.3 (C_6_H_5_), 124.3 (^Aza^C), 113.8 (^Aza^CH), 100.4 (^Aza^CH), 1.04 (BCH). ^31^P{^1^H} NMR (δ, C_6_D_6_): 1.17. ^11^B NMR (δ, C_6_D_6_): −6.24 (s, h.h.w. = 184 Hz). ^11^B{^1^H} NMR (δ, C_6_D_6_): −6.24 (s, h.h.w. = 121 Hz). IR (cm^−1^, powder film): 2120, 2062. MS ES^+^ (*m*/*z*): 649 [Cu(**^Ph^Bai**)PPh_3_]^+^.

### 3.3. Synthesis of Synthesis of [Cu{κ^3^-N,N,H-**^Naphth^Bai**}(PPh_3_)] (**3**)

A clean dry Schlenk flask was charged with CuCl (44 mg, 0.45 mmol), ligand precursor [**Li**(**^Naphth^Bai**)**]** (200 mg, 0.45 mmol) and PPh_3_ (112 mg, 0.45 mmol). Methanol (10 mL) was subsequently added, and the reaction mixture was stirred for 2 h at room temperature. The solvent was then removed via filtration. The precipitate was washed with MeCN (2 × 5 mL) and dried to give [Cu(**^Naphth^Bai**)(PPh_3_)] as an off-white solid (243 mg, 0.34 mmol, 79%). NMR δ ppm: ^1^H (C_6_D_6_, 400 MHz), 8.20 (d, ^3^J_HH_ = 6.49 Hz, 1H, ^Naphth^CH), 8.10 (d, ^3^J_HH_ = 8.34 Hz, 1H, ^Naphth^CH), 7.97 (d, ^3^J_HH_ = 4.63 Hz, 2H, ^Aza^CH), 7.81 (d, ^3^J_HH_ = 8.34 Hz, 2H, ^Aza^CH), 7.67 (s, 2H, ^Aza^CH), 7.51–7.58 (m, 8H, P(C_6_H_5_) + ^Naphth^CH), 7.49 (t, ^3^J_HH_ = 7.32 Hz, 1H, ^Naphth^CH), 7.22 (t, ^3^J_HH_ = 7.42 Hz, 1H, ^Naphth^CH), 7.15 (s, 1H, ^Naphth^CH), 6.97 (unresolved, H, BH), 6.81–6.97 (m, 9H, P(C_6_H_5_) + ^Naphth^CH), 6.54 (dd, ^3^J_HH_ = 2.55 Hz, ^3^J_HH_ = 5.1 Hz, 2H, ^Aza^CH), 6.35 (s, 2H, ^Aza^CH). ^13^C {^1^H} (C_6_D_6_, 100 MHz), 151.6 (^Aza^C), 143.1 (^Naphth^C), 140.4 (^Aza^CH), 137.7 (^Naphth^C), 134.4 (^Aza^C), 133.6 (d, ^2^J_CP_ = 15.4 Hz, P^ortho^(C_6_H_5_)), 133.6 (d, ^1^J_CP_ = 27.5 Hz, P^ipso^(C_6_H_5_)), 132.0 (^Naphth^CH), 129.6 (^Naphth^CH), 128.7 (d, ^2^J_CP_ = 10.6 Hz, P^meta^(C_6_H_5_)), 128.2 (^Naphth^CH), 128.6 (d, ^2^J_CP_ = 5.7 Hz, P^para^(C_6_H_5_)), 127.0 (^Naphth^CH), 125.3 (^Aza^CH), 124.4 (^Naphth^C), 124.4 (^Naphth^C), 113.9 (^Aza^CH), 100.5 (^Aza^CH), 1.08 (BCH). ^31^P{^1^H} NMR (δ, C_6_D_6_): 1.64. ^11^B NMR (δ, C_6_D_6_): −6.23 (s, h.h.w. = 180 Hz). ^11^B{^1^H} NMR (δ, C_6_D_6_): −6.23 (s, h.h.w. = 133 Hz). IR (cm^−1^, powder film): 2125, 2082. MS ES^+^ (*m*/*z*): 699 [Cu(**^Naphth^Bai**)PPh_3_]^+^, 687 [Cu(Naphth)(7-azaindolyl)_2_PPh_3_)].

### 3.4. Synthesis of [Cu{κ^3^-N,N,H-**^Me^Bai**}(PCy_3_)] (**4**)

A clean dry Schlenk flask was charged with CuCl (100 mg, 1.00 mmol), ligand precursor [**Li**(**^Me^Bai**)**]** (271 mg, 1.00 mmol) and PCy_3_ (283 mg, 1.00 mmol). Methanol (20 mL) was subsequently added, and the reaction mixture was stirred for 2 h at room temperature. The solvent was then removed via filtration to give the precipitate [Cu(**^Me^Bai**)(PCy_3_)] as a white solid, which was washed with acetonitrile and dried (436 mg, 0.72 mmol, 72%). NMR δ ppm: ^1^H (C_6_D_6_, 400 MHz), 8.27 (dd, ^3^J_HH_ = 3.61 Hz, ^3^J_HH_ = 1.40 Hz, 2H, ^Aza^CH), 7.83 (dd, ^3^J_HH_ = 6.12 Hz, ^3^J_HH_ = 1.53 Hz, 2H, ^Aza^CH), 7.59 (d, ^3^J_HH_ = 3.25 Hz, 2H, ^Aza^CH), 6.69 (dd, ^3^J_HH_ = 2.60 Hz, ^3^J_HH_ = 5.04 Hz, 2H, ^Aza^CH), 6.41 (d, ^3^J_HH_ = 3.16 Hz, 2H, ^Aza^CH), 5.49 (unresolved, H, BH), 1.19–1.92 (m, 30H, P(C_6_H_11_)), 1.17 (broad doublet, 3H, BCH_3_, apparent J_HH_ = 4.31 Hz, this signal became more resolved in the corresponding ^1^H{^11^B} experiment, ^3^J_HH_ = 4.41 Hz), ^13^C {^1^H} (CDCl_3_, 100 MHz), 150.3 (^Aza^CH), 140.2 (^Aza^CH), 131.1 (^Aza^CH), 127.9 (^Aza^CH), 123.7 (^Aza^CH), 113.4 (^Aza^CH), 99.0 (^Aza^CH), 32.7 (d, ^3^J_CP_ = 15.37 Hz, P(C_6_H_11_)), 31.06 (d, ^3^J_CP_ = 2.3 Hz, P(C_6_H_11_)), 30.36 (d, ^3^J_CP_ = 3.98 Hz, P(C_6_H_11_)), 27.6 (d, ^3^J_CP_ = 10.70 Hz, P(C_6_H_11_)), 26.3 (BCH_3_). ^31^P{^1^H} NMR (δ, C_6_D_6_): 17.74. ^11^B NMR (δ, C_6_D_6_): −7.65 (s, h.h.w. = 181 Hz). ^11^B{^1^H} NMR (δ, C_6_D_6_): −7.65 (s, h.h.w. = 123 Hz). IR (cm^−1^, powder film): 2141, 2087. MS ES^+^ (*m*/*z*): the main peak here was 623.4, which corresponded to [Cu(PCy_3_)_2_]^+^.

### 3.5. Synthesis of [Cu{κ^3^-N,N,H-**^Ph^Bai**}(PCy_3_)] (**5**)

A clean dry Schlenk flask was charged with CuCl (100 mg, 1.00 mmol), ligand precursor [**Li**(**^Ph^Bai**)**]** (330 mg, 1.00 mmol) and PCy_3_ (280 mg, 1.00 mmol). Methanol (20 mL) was subsequently added, and the reaction mixture was stirred for 2 h at room temperature. The solvent was then removed via filtration to give the precipitate [Cu(**^Ph^Bai**)(PCy_3_)] as a white solid (603 mg, 0.90 mmol, 90%). NMR δ ppm: ^1^H (C_6_D_6_, 400 MHz), 8.28 (d, ^3^J_HH_ = 5.08 Hz, 2H, ^Aza^CH), 7.90 (d, ^3^J_HH_ = 6.84 Hz, 2H, C_6_H_5_), 7.83 (d, ^3^J_HH_ = 3.35 Hz, 2H, ^Aza^CH), 7.58 (d, ^3^J_HH_ = 7.71 Hz, 2H, C_6_H_5_), 7.45 (t, ^3^J_HH_ = 7.49 Hz, 2H, ^Aza^CH), 7.31 (t, ^3^J_HH_ = 7.33 Hz, H, C_6_H_5_), 6.70 (dd, ^3^J_HH_ = 5.30 Hz, 2H, ^Aza^CH), 6.40 (d, ^3^J_HH_ = 3.18 Hz, 2H, ^Aza^CH), 6.36 (unresolved, H, BH), 1.28–1.86 (m, 18H, P(C_6_H_11_)), 0.91–1.12 (m, 12H, P(C_6_H_11_)), ^13^C {^1^H} (C_6_D_6_, 100 MHz), 150.7 (^Aza^C), 139.1 (^Aza^CH), 132.9 (C_6_H_5_), 132.3 (^Aza^CH), 127.6 (^Aza^CH), 126.9 (C_6_H_5_), 126.7 (C_6_H_5_), 126.5 (C_6_H_5_), 124.4 (C_6_H_5_), 123.6 (^Aza^CH), 112.8 (^Aza^CH), 99.5 (^Aza^CH), 31.8 (d, ^2^J_CP_ = 15.1 Hz, P(C_6_H_11_)), 29.6 (s, P(C_6_H_11_)), 29.4 (d, ^4^J_CP_ = 4.1 Hz, P(C_6_H_11_)), 26.5 (d, ^3^J_CP_ = 10.7 Hz, P(C_6_H_11_)), 0.21 (BCH). ^31^P{^1^H} NMR (δ, C_6_D_6_): 18.08. ^11^B NMR (δ, C_6_D_6_): −6.37 (s, h.h.w. = 175 Hz). ^11^B{^1H^} NMR (δ, C_6_D_6_): −6.37 (s, h.h.w. = 129 Hz). IR (cm^−1^, powder film): 2169, 2076. Elemental analysis calc. for C_38_H_49_BCuN_4_P•MeOH (%): C 66.90, H 7.77, N 8.00; found: C 66.30, H 7.16, N 8.59. MS ES^+^ (*m*/*z*): 667 [Cu(**^Ph^Bai**)PCy_3_]^+^_,_ 710.2 [Cu(**^Ph^Bai**)_2_]^+^.

### 3.6. Synthesis of [Cu{κ^3^-N,N,H-**^Naphth^Bai**}(PCy_3_)] (**6**)

A clean dry Schlenk flask was charged with CuCl (44 mg, 0.45 mmol), ligand precursor [Li(**^Naphth^Bai**)] (200 mg, 0.45 mmol) and PCy_3_ (120 mg, 0.45 mmol). Methanol (10 mL) was subsequently added and the reaction mixture was stirred for 2 h at room temperature. The solvent was then removed via filtration. The precipitate was washed with MeCN (2 × 5 mL) to give [Cu(**^Naphth^Bai**)(PCy_3_)] as an off-white solid (243 mg, 0.34 mmol, 79%). NMR δ ppm: ^1^H (C_6_D_6_, 400 MHz), 8.30 (d, ^3^J_HH_ = 5.03 Hz, 2H, ^Aza^CH), 8.20 (d, ^3^J_HH_ = 6.82 Hz, 1H, Naphth-CH), 8.08 (d, ^3^J_HH_ = 8.60 Hz, 1H, Naphth-CH), 7.82 (t, ^3^J_HH_ = 8.04 Hz, 2H, Naphth-CH), 7.65 (d, ^3^J_HH_ = 3.20 Hz, 2H, ^Aza^CH), two overlapping peaks: 7.56 (d, ^3^J_HH_ = 7.69 Hz, 2H, ^Aza^CH), 7.55 (t, ^3^J_HH_ = 7.79 Hz, 1H, Naphth-CH), 7.29 (t, ^3^J_HH_ = 7.76 Hz, 1H, Naphth-CH), 6.72 (unresolved, 1H, BH), 6.70 (dd, ^3^J_HH_ = 5.09 Hz, 2H, ^Aza^CH), 6.34 (d, ^3^J_HH_ = 3.31 Hz, 2H, ^Aza^CH), 1.71–1.85 (m, 8H, P(C_6_H_11_)), 1.27–1.44 (m, 14H, P(C_6_H_11_)), 0.81–0.98 (m, 8H, P(C_6_H_11_)), remaining naphthalene proton peaks lie under the solvent peak at 7.26 ppm. ^13^C{^1^H} (C_6_D_6_, 100 MHz), 150.9 (^Aza^C), 140.1 (^Aza^CH), 137.8 (^Naphth^C), 134.5 (^Naphth^C), 133.5 (^Aza^CH), 131.8 (^Naphth^CH), 129.1 (^Naphth^CH), 128.6 (^Naphth^CH), 128.5 (^Aza^CH), 128.1 (^Naphth^CH), 126.8 (^Naphth^CH), 125.3 (^Naphth^CH), 124.3 (^Aza^C), 124.2 (^Naphth^CH), 124.1 (^Naphth^CH), 113.7 (^Aza^CH), 100.5 (^Aza^CH), 32.5 (d, ^3^J_CP_ = 17.08 Hz, P(C_6_H_11_)), 30.3 (d, ^3^J_CP_ = 3.99 Hz, P(C_6_H_11_)), 29.7 (s, P(C_6_H_11_)), 27.2 (d, ^3^J_CP_ = 10.62 Hz, P(C_6_H_11_)), 1.1 (s, BCH_3_). ^31^P{^1^H} NMR (δ, C_6_D_6_): 18.63. ^11^B NMR (δ, C_6_D_6_): −6.96 (s, h.h.w. = 213 Hz). ^11^B{^1H^} NMR (δ, C_6_D_6_): −6.96 (s, h.h.w. = 123 Hz). IR (cm^−1^, powder film): 2189, 2090. MS ES^+^ (*m*/*z*): 717 [Cu(**^Naphth^Bai**)PCy_3_]^+^.

### 3.7. Synthesis of [Cu{κ^3^-N,N,H-**^Me^Bai**}_2_] (**7**)

Copper(II) chloride (19 mg, 0.14 mmol) and [Li(**^Me^Bai**)] (100 mg, 0.29 mmol) were placed into a Schlenk flask, along with methanol (10 mL) under an inert atmosphere. The suspension was stirred for 2 h at room temperature. Subsequently, the supernatant was filtered off and the precipitate was washed with MeOH (10 mL) and MeCN (10 mL) to give a dark yellow solid (91%, 73 mg, 0.13 mmol). IR (cm^−1^, powder film): 2226. MS ES^+^ (*m*/*z*): 586.2 [Cu(**^Me^Bai**)_2_]^+^.

### 3.8. Synthesis of [Cu{κ^3^-N,N,H-**^Ph^Bai**}_2_] (**8**)

Copper(II) chloride (16 mg, 0.12 mmol) and [Li(**^Ph^Bai**)] (100 mg, 0.24 mmol) were put into a Schlenk flask, and methanol (10 mL) was added under an inert atmosphere. The suspension was stirred for 2h at room temperature. Subsequently, the supernatant was filtered off and the precipitate was washed with MeOH (10 mL) and MeCN (10 mL) to give a dark yellow solid (80%, 68 mg, 0.1 mmol). IR (cm^−1^, powder film): 2269. Elemental analysis calc. for C_40_H_32_B_2_CuN_8_•½ MeOH (%): C 67.01, H 4.72, N 15.44; found: C 66.80, H 4.37, N 15.13. MS ES^+^ (*m*/*z*): 710.2 [Cu(**^Ph^Bai**)_2_]^+^, 772.2 [Cu_2_(**^Ph^Bai**)_2_ − H]^+^.

### 3.9. Synthesis of [Cu{κ^3^-N,N,H-**^Naphth^Bai**}_2_] (**9**)

Copper(II) chloride (15 mg, 0.11 mmol) and [Li(**^Naphth^Bai**)] (100 mg, 0.22 mmol) were put into a Schlenk flask, and methanol (10 mL) was added under an inert atmosphere. The suspension was stirred for 2 h at room temperature. Subsequently, the supernatant was filtered off and the precipitate was washed with MeOH (10 mL) and MeCN (10 mL) to give a dark yellow solid (75 %, 67 mg, 0.08 mmol). IR (cm^−1^, powder film): 2221. Elemental analysis calc. for C_48_H_36_B_2_CuN_8_•MeOH (%): C 69.89, H 4.79, N 13.31; found: C 70.14, H 4.39, N 13.38. MS ES^+^ (*m*/*z*): 809.2 [Cu(**^Naphth^Bai**)_2_]^+^.

## 4. Conclusions

The synthesis and characterization of a series of copper(I) and copper(II) complexes containing the novel [**^R^Bai**]^−^ scorpionate ligands are reported herein. Eight of the nine new complexes were structurally characterized via X-ray crystallography. In all cases, a κ^3^-*N*,*N*,*H* coordination mode, where both 7-azaindolyl “arms” were coordinated, along with the BH unit, was observed. The crystal structures confirmed significant B–H---Cu interactions. This was supported by solid-state infrared spectroscopy, where the structures were maintained, to some degree, in solution, as evidenced by multinuclear spectroscopy data. Furthermore, the structural characterization highlights a high degree of flexibility in terms of the size of the N–M–N angles and the approach of the B–H unit within the **^R^Bai** ligands upon coordination.

These new copper(I) and copper(II) complexes add to the family of complexes containing flexible 7-azaindole-based scorpionate ligands, which, despite being first reported in 2005, are still underdeveloped. This investigation consolidated the fact that the κ^3^-*N*,*N*,*H* coordination mode is the preferred mode of binding. The crystal structures demonstrated flexibility in the N–M–N chelating angles. This had an impact on the positioning of the B–H---M interaction. The potential of the azaindolyl-based “flexible scorpionates” in terms of reactivity and applications has yet to be fully realized. The reactivity of the BH unit with the metal center is likely to be instrumental in defining this reactivity. These aspects are currently under investigation.

## Figures and Tables

**Table 1:** Selected NMR (ppm; Hz) and IR (cm^−1^) spectroscopic data for [**^R^Bai**] ligand salt precursors and their corresponding copper complexes. The solvent used for the NMR studies was C_6_D_6_.

Ligand/Complex	^31^P{^1^H} NMR	^11^B{^1^H} NMR	h.h.w in ^11^B/^11^B{^1^H}	^1^H{^11^B} NMR B-H	IR (B-H) Powder Film
Li[**^Me^Bai**]	-	−8.14	-	4.66	2396, 2272
Li[**^Ph^Bai**]	-	−6.80	-	5.47	2394, 2264
Li[**^Naphth^Bai**]	-	−6.87	-	6.05	2429, 2272
[Cu(**^Me^Bai**)(PPh_3_)] (**1**)	1.41	−7.27	186/129	5.63	2143, 2089
[Cu(**^Ph^Bai**)(PPh_3_)] (**2**)	1.17	−6.24	184/121	6.58	2120, 2062
[Cu(**^Naphth^Bai**)(PPh_3_)] (**3**)	1.64	−6.23	180/133	6.97	2125, 2082
[Cu(**^Me^Bai**)(PCy_3_)] (**4**)	17.74	−7.65	181/123	5.49	2141, 2087
[Cu(**^Ph^Bai**)(PCy_3_)] (**5**)	18.08	−6.37	175/129	6.36	2169, 2076
[Cu(**^Naphth^Bai**)(PCy_3_)] (**6**)	18.63	−6.96	213/123	6.72	2189, 2090

**Table 2:** Selected distances (Å) and angles (°) for **1**–**3** along with a comparison with [Cu(**Tai**)(PPh_3_)].

Distance (Å)/Angle (°)	1	2	3 • (MeCN)	Cu(Tai)(PPh_3_) ^a^
Cu(1)–N(2)/Cu(1)–N(4)	2.0228(5)/2.0174(5)	2.0259(5)/2.0206(6)	2.0350(8)/2.0477(9)	2.008(3)/2.009(3)
B(1)–N(1)/B(1)–N(3)	1.5507(9)/1.5652(9)	1.5522(9)/1.5598(10)	1.5561(14)/1.5567(14)	1.558(5)/1.547(4)/*1.550(4)* ^b^
B(1)–C(15)	1.6106(9)	1.6127(11)	1.6181(16)	-
Cu(1)–P(1)	2.18341(17)	2.1825(2)	2.1899(3)	2.1844(12)
Cu(1)∙∙∙B(1)	2.7757(7)	2.7741(8)	2.7860(14)	2.808(3)
B(1)–H(1)	1.230(8)	1.268(9)	1.282(12)	1.20(3)
Cu(1)∙∙∙H(1)	1.847(8)	1.813(9)	1.803(11)	1.81(3)
N(2)–Cu(1)–P(1)	125.192(16)	118.485(15)	118.79(3)	125.31(8)
N(4)–Cu(1)–P(1)	123.969(17)	121.888(19)	124.43(3)	126.73(9)
N(4)–Cu(1)–N(2)	107.73(2)	108.70(2)	107.27(3)	105.87(12)
H(1)–Cu(1)–P(1)	104.7(2)	119.5(3)	118.3(3)	107(1)
H(1)–Cu(1)–N(2)	90.7(2)	88.9(2)	88.1(3)	88(1)
H(1)–Cu(1)–N(4)	90.6(2)	92.0(3)	91.1(3)	86(1)
∑ of angles of non-hydrogen substituents at copper	356.89(3)	349.07(3)	350.49(5)	357.91(17)
N(1)–B(1)–N(3)	109.77(5)	109.42(6)	106.89(8)	111.7(3)
C(15)–B(1)–N(1)	110.77(5)	114.67(6)	114.94(9)	*113.7(2)* ^b, c^
C(15)–B(1)–N(3)	112.35(5)	110.53(5)	111.63(9)	*111.8(3)* ^b, c^
∑ of angles of non-hydrogen substituents at boron	332.89(9)	334.62(9)	333.46(15)	337.2(5)
B–H∙∙∙Cu(1)	127.7(5)	127.6(6)	128.3(7)	136(2)
Cu(1)–N–N–B(1) ^d^	−0.75(4)/−2.07(4)	−4.72(4)/−1.55(4)	7.07(6)/12.13(6)	−5.7(2)/−7.8(2)
Position of the Ar group at B ^e^	-	64.48(8)	57.66(14)	178.6(2) ^b, c^

^a^ The corresponding values for this structure were obtained from the published article or by measurements obtained from the published cif file using Mercury software; ^b^ the values in italics are those that corresponded to the uncoordinated 7-azaindolyl “arm” in the **Tai** ligand; ^c^ the values corresponded to a nitrogen atom in the third 7-azaindolyl unit in **Tai** rather than the corresponding carbon atoms in **^R^Bai**; ^d^ the two torsion angles involving the relative positions of the copper and boron atoms about the two nitrogen atoms of the coordinated 7-azaindolyl rings; ^e^ defined by the modulus of the smallest torsion angle M--B–C_ipso_–C_ortho_ for **^R^Bai** or M--B–N--N for **Tai**.

**Table 3:** Selected distances (Å) and angles (°) for **4**–**6**.

Distance (Å)/Angle (°)	4	5 ^a^	6 • (MeCN)
Cu(1)–N(2)/Cu(1)–N(4)	2.0740(16)/1.9955(16)	2.034(3)/2.032(3)	2.0384(4)/2.0515(4)
B(1)–N(1)/B(1)–N(3)	1.560(3)/1.565(3)	1.555(5)/1.552(5)	1.5646(7)/1.5545(7)
B(1)–C(15)	1.603(3)	1.620(6)	1.6161(7)
Cu(1)–P(1)	2.1802(5)	[2.205(3)]/[2.208(3)] ^a^	2.19484(15)
Cu(1)∙∙∙B(1)	2.763(2)	2.776(4)	2.7995(7)
B(1)–H(1)	1.23(2)	1.05(6)	1.243(7)
Cu(1)∙∙∙H(1)B(1)	1.85(2)	1.92(6)	1.847(6)
N(2)–Cu(1)–P(1)	118.87(5)	[118.17(11)]/[129.61(11)] ^a^	126.875(13)
N(4)–Cu(1)–P(1)	137.38(5)	[129.65(11)]/[118.19(11)] ^a^	123.195(13)
N(4)–Cu(1)–N(2)	99.77(6)	109.95(12)	104.488(17)
H(1)–Cu(1)–P(1)	105.1(7)	[109.1(18)]/[109.1(18)] ^a^	112.0(2)
H(1)–Cu(1)–N(2)	93.4(6)	86.9(17)	88.0(2)
H(1)–Cu(1)–N(4)	88.7(7)	86.7(17)	90.1(2)
∑ of angles of non-hydrogen substituents at copper	356.02(9)	[357.7(2)]/[357.7(2)] ^a^	354.56(3)
N(1)–B(1)–N(3)	110.42(16)	110.6(3)	107.81(4)
C(15)–B(1)–N(1)	111.14(15)	112.0(3)	113.59(4)
C(15)–B(1)–N(3)	111.19(17)	112.1(3)	112.34(4)
∑ of angles of non-hydrogen substituents at boron	332.8(3)	334.7(5)	333.74(7)
B–H∙∙∙Cu(1)	127.0(16)	136(5)	128.8(5)
Cu(1)–N–N–B(1) ^b^	12.24(11)/20.97(11)	1.7(2)/−1.7(2)	−7.41(3)/−8.30(3)
Position of the Ar group at B ^c^	-	87.5(4)	70.00(6)

^a^ The structure for complex **5** showed disorder with the PCy_3_ ligand over two positions (ca. 50:50), and thus, there are two values for all measurements involving P(1); ^b^ the two torsion angles that involved the relative positions of the copper and boron atoms about the two nitrogen atoms of the coordinated 7-azaindolyl rings; ^c^ defined by the modulus of the smallest torsion angle M--B–C_ipso_–C_ortho_.

**Table 4:** Selected distances (Å) and angles (°) for **7** and **8**.

Distance (Å)/Angle (°)	7	8 • (CH_2_Cl_2_) ^a^
Cu(1)–N(2)/Cu(1)–N(4)	2.0161(19)/2.0316(16)	[2.0455(13)/2.0235(12)]/[2.0358(12)/2.0159(12)]
B(1)–N(1)/B(1)–N(3)	1.560(3)/1.553(3)	[1.547(2)/1.554(2)]/[1.5478(19)/1.554(2)]
B(1)–C(15)	1.606(3)	[1.599(2)]/[1.603(2)]
Cu(1)∙∙∙B(1)	2.944(2)	[2.9384(16)]/[2.9281(15)]
B(1)–H(1)	1.18(2)	[1.16(2)]/[1.14(2)]
Cu(1)∙∙∙H(1)B(1)	2.08(2)	[2.00(2)]/[2.03(2)]
N(2)–Cu(1)–N(4)	89.77(6)	[92.94(5)]/[91.10(5)]
N(2)`–Cu(1)–N(4)	90.23(6)	[87.06(5)]/[88.90(5)]]
H(1)–Cu(1)–N(2)	87.6(6)	[85.0(6)]/[85.1(6)]
H(1)–Cu(1)–N(4)	88.1(6)	[85.3(6)]/[86.3(6)]
N(1)–B(1)–N(3)	107.99(16)	[108.88(12)]/[108.58(11)]
C(15)–B(1)–N(1)	111.40(17)	[112.15(12)]/[114.06(11)]
C(15)–B(1)–N(3)	111.98(16)	[115.15(12)]/[112.51(11)]
∑ of angles of non-hydrogen substituents at boron	331.4(3)	[336.2(2)]/[335.15(19)]
B–H∙∙∙Cu(1)	126.5(16)	[135.2(15)]/[133.7(15)]
Cu(1)–N–N–B(1) ^b^	0.31(11)/−16.58(12)	[5.73(9)/−7.65(9)]/[−9.25(8)/6.28(8)]
Position of the Ar group at B ^c^	-	[77.96(15)]/[78.03(15)]

^a^ There were two independent complexes of equal occupancy within this structure, and thus, two sets of values are provided in the table. The values for each complex are separated by square parenthesis. ^b^ The two torsion angles that involved the relative positions of the copper and boron atoms about the two nitrogen atoms of the coordinated 7-azaindolyl rings. ^c^ Defined by the modulus of the smallest torsion angle M--B–C_ipso_–C_ortho_.

## Supplementary Materials

The following supporting information can be downloaded at: https://www.mdpi.com/article/10.3390/molecules28124825/s1, which contains details on the crystallographic information parameters (Tables S1–S3), as well as crystallographic collection and refinement details and selected NMR spectra for complexes **1**–**6** (Figures S1.1–S6.4).
