# Supplementary material for: Synthesis and Structural Characterization of Copper Complexes Containing “R-Substituted” Bis-7-Azaindolyl Borate Ligands
Source: Molecules. 2023 Jun 17;28(12):4825. doi: 10.3390/molecules28124825 (PMC10303152; doi:10.3390/molecules28124825)
Supplement: Supplementary file 1 [file molecules-28-04825-s001.zip › molecules-2432312-supplementary.pdf]

Supplementary Materials for:

Synthesis and Structural Characterization of Copper Complexes Containing "R-substituted" Bis-7-Azaindolyl Borate Ligands

Miriam Jackson [1], Simon D. Thomas [1], Graham J. Tizzard [2], Simon J. Coles [2] and Gareth R. Owen [1,*]

[1] Chemical and Environmental Sciences, Faculty of Computing, Engineering and Science, University of South Wales, Pontypridd CF37 4AT, UK; miriam.jackson@southwales.ac.uk (M.J.); simon_thomas@live.com (S.D.T.)

[2] UK National Crystallography Service, School of Chemistry, University of Southampton, Highfield, Southampton SO17 1BJ, UK; graham.tizzard@soton.ac.uk (G.J.T.); s.j.coles@soton.ac.uk (S.J.C.)

* Correspondence: gareth.owen@southwales.ac.uk; Tel.: +44-1443-65-4527

Contents

A - Crystallography

A1. **Crystallographic Information for Complexes 1 - 8 (Page S3)**
Table S1. Important X-ray data collection parameters for the [Cu(^{R}**Bai**)PPh$_3$)] complexes, **1** - **3**.
Table S2. Important X-ray data collection parameters for the [Cu(^{R}**Bai**)PCy$_3$)] complexes, **4** - **6**.
Table S3. Important X-ray data collection parameters for the [Cu(^{R}**Bai**)$_2$)] complexes, **7** and **8**.

A2 **Crystallographic data collection, reduction, and structure solution refinement (Page S6)**

B - Selected NMR Spectra and Mass Spectrometry Data

B1 **Spectra for [Cu{κ^3-*N,N,H*-^{Me}Bai}(PPh$_3$)] (1) (Page S10)**
Fig S1.1: ^{1}H{^{11}B} NMR spectrum of the complex **[Cu{κ^3-*N,N,H*-^{Me}Bai}(PPh$_3$)]** C_6D_6 at 298 K.
Fig S1.2: ^{13}C NMR spectrum of the complex **[Cu{κ^3-*N,N,H*-^{Me}Bai}(PPh$_3$)]** C_6D_6 at 298 K.
Fig S1.3: ^{31}P{^{1}H} NMR spectrum of the complex **[Cu{κ^3-*N,N,H*-^{Me}Bai}(PPh$_3$)]** C_6D_6 at 298 K.
Fig S1.4: ^{11}B{^{1}H} NMR spectrum of the complex **[Cu{κ^3-*N,N,H*-^{Me}Bai}(PPh$_3$)]** C_6D_6 at 298 K.
Fig S1.5: Mass spectrometry data for **[Cu{κ^3-*N,N,H*-^{Me}Bai}(PPh$_3$)]**

B2 **Spectra for [Cu{κ^3-*N,N,H*-^{Ph}Bai}(PPh$_3$)] (2) (Page S13)**
Fig S2.1: ^{1}H{^{11}B} NMR spectrum of the complex **[Cu{κ^3-*N,N,H*-^{Ph}Bai}(PPh$_3$)]** C_6D_6 at 298 K.
Fig S2.2: ^{13}C NMR spectrum of the complex **[Cu{κ^3-*N,N,H*-^{Ph}Bai}(PPh$_3$)]** C_6D_6 at 298 K.
Fig S2.3: ^{31}P{^{1}H} NMR spectrum of the complex **[Cu{κ^3-*N,N,H*-^{Ph}Bai}(PPh$_3$)]** C_6D_6 at 298 K.
Fig S2.4: ^{11}B{^{1}H} NMR spectrum of the complex **[Cu{κ^3-*N,N,H*-^{Ph}Bai}(PPh$_3$)]** C_6D_6 at 298 K
Fig S2.5: Mass spectrometry data for **[Cu{κ^3-*N,N,H*-^{Ph}Bai}(PPh$_3$)]**

B3 **Spectra for [Cu{κ^3-*N,N,H*-^{Naphth}Bai}(PPh$_3$)] (3) (Page S16)**
Fig S3.1: ^{1}H{^{11}B} NMR spectrum of the complex **[Cu{κ^3-*N,N,H*-^{Naphth}Bai}(PPh$_3$)]** C_6D_6 at 298 K.
Fig S3.2: ^{13}C NMR spectrum of the complex **[Cu{κ^3-*N,N,H*-^{Naphth}Bai}(PPh$_3$)]** C_6D_6 at 298 K.
Fig S3.3: ^{31}P{^{1}H} NMR spectrum of the complex **[Cu{κ^3-*N,N,H*-^{Naphth}Bai}(PPh$_3$)]** C_6D_6 at 298 K.
Fig S3.4: ^{11}B{^{1}H} NMR spectrum of the complex **[Cu{κ^3-*N,N,H*-^{Naphth}Bai}(PPh$_3$)]** C_6D_6 at 298 K.
Fig S3.5: Mass spectrometry data for **[Cu{κ^3-*N,N,H*-^{Naphth}Bai}(PPh$_3$)]**

B4 **Spectra for [Cu{κ^3-*N,N,H*-^{Me}Bai}(PCy$_3$)] (4) (Page S19)**
Fig S4.1: ^{1}H{^{11}B} NMR spectrum of the complex **[Cu{κ^3-*N,N,H*-^{Me}Bai}(PCy$_3$)]** C_6D_6 at 298 K.
Fig S4.2: ^{13}C NMR spectrum of the complex **[Cu{κ^3-*N,N,H*-^{Me}Bai}(PCy$_3$)]** C_6D_6 at 298 K.
Fig S4.3: ^{31}P{^{1}H} NMR spectrum of the complex **[Cu{κ^3-*N,N,H*-^{Me}Bai}(PCy$_3$)]** C_6D_6 at 298 K.

Fig S4.4: ^{11}B{^{1}H} NMR spectrum of the complex **[Cu{κ^{3}-*N,N,H*-^{Me}Bai}(PCy$_3$)]** C_6D_6 at 298 K.
Fig 4.5: Mass spectrometry data for **[Cu{κ^{3}-*N,N,H*-^{Me}Bai}(PCy$_3$)]**

B5 Spectra for [Cu{κ^{3}-*N,N,H*-^{Ph}Bai}(PCy$_3$)] (5) (Page S22)
Fig S5.1: ^{1}H{^{11}B} NMR spectrum of the complex **[Cu{κ^{3}-*N,N,H*-^{Ph}Bai}(PCy$_3$)]** C_6D_6 at 298 K.
Fig S5.2: ^{13}C NMR spectrum of the complex **[Cu{κ^{3}-*N,N,H*-^{Ph}Bai}(PCy$_3$)]** C_6D_6 at 298K.
Fig S5.3: ^{31}P{^{1}H} NMR spectrum of the complex **[Cu{κ^{3}-*N,N,H*-^{Ph}Bai}(PCy$_3$)]** C_6D_6 at 298 K.
Fig S5.4: ^{11}B{^{1}H} NMR spectrum of the complex **[Cu{κ^{3}-*N,N,H*-^{Ph}Bai}(PCy$_3$)]** C_6D_6 at 298 K.
Fig S5.5: Mass spectrometry data for **[Cu{κ^{3}-*N,N,H*-^{Ph}Bai}(PCy$_3$)]**

B6 Spectra for [Cu{κ^{3}-*N,N,H*-^{Naphth}Bai}(PCy$_3$)] (6) (Page S25)
Fig S6.1: ^{1}H{^{11}B} NMR spectrum of the complex **[Cu{κ^{3}-*N,N,H*-^{Naphth}Bai}(PCy$_3$)]** C_6D_6 at 298 K.
Fig S6.2: ^{13}C NMR spectrum of the complex **[Cu{κ^{3}-*N,N,H*-^{Naphth}Bai}(PCy$_3$)]** C_6D_6 at 298 K.
Fig S6.3: ^{31}P{^{1}H} NMR spectrum of the complex **[Cu{κ^{3}-*N,N,H*-^{Naphth}Bai}(PCy$_3$)]** C_6D_6 at 298 K.
Fig S6.4: ^{11}B{^{1}H} NMR spectrum of the complex **[Cu{κ^{3}-*N,N,H*-^{Naphth}Bai}(PCy$_3$)]** C_6D_6 at 298 K.
Fig S6.5: Mass spectrometry data for **[Cu{κ^{3}-*N,N,H*-^{Naphth}Bai}(PCy$_3$)]**

B7 Spectra for [Cu{κ^{3}-*N,N,H*-^{Me}Bai}$_2$] (7) (Page S28)
Fig S7.1: Mass spectrometry data for **[Cu{κ^{3}-*N,N,H*-^{Me}Bai}$_2$]**

B8 Spectra for [Cu{κ^{3}-*N,N,H*-^{Ph}Bai}$_2$] (8) (Page S28)
Fig S8.1: Mass spectrometry data for **[Cu{κ^{3}-*N,N,H*-^{Ph}Bai}$_2$]**

B9 Spectra for [Cu{κ^{3}-*N,N,H*-^{Naphth}Bai}$_2$] (9) (Page S29)
Fig S9.1: Mass spectrometry data for **[Cu{κ^{3}-*N,N,H*-^{Naphth}Bai}$_2$]**

A1. Crystallographic Information for Complexes 1 - 8

Table S1. Important X-ray data collection parameters for the [Cu(^{R}**Bai**)PPh_3)] complexes, **1** - **3**.

**Compound**	**[Cu(^{Me}Bai)PPh_3] (1)**	**[Cu(^{Ph}Bai)PPh_3] (2)**	**[Cu(^{Naphth}Bai)PPh_3] (3)** [a]
CCDC No:	2263427	2263428	22634279
NCS No:	2019ncsx0289z	2022ncs0565z	2022ncs0364
Formula	$C_{33}H_{29}BCuN_4P$	$C_{38}H_{31}BCuN_4P$	$C_{44}H_{36}BCuN_5P$
$D_{calc.}$/g cm^{-3}	1.366	1.375	1.359
m/mm^{-1}	0.851	0.782	0.688
Formula Weight	586.969	649.040	740.153
Colour	colourless	colourless	colourless
Shape	prism-shaped	rod-shaped	needle-shaped
Size/mm^3	0.20×0.14×0.09	0.22×0.02×0.02	0.30×0.02×0.01
T/K	100(2)	100.00(10)	100(2)
Crystal System	monoclinic	monoclinic	monoclinic
Space Group	$P2_1/c$	$P2_1$	$P2_1/c$
a/Å	11.0091(1)	9.0350(2)	17.4959(5)
b/Å	18.1167(2)	14.9601(3)	8.9216(3)
c/Å	14.7128(2)	12.0788(2)	23.3070(9)
a/°	90	90	90
b/°	103.421(1)	106.238(2)	96.074(3)
g/°	90	90	90
V/Å^3	2854.31(6)	1567.50(6)	3617.6(2)
Z	4	2	4
Z'	1	1	1
Wavelength/Å	0.71073	0.71075	0.71075
Radiation type	Mo K_a	Mo K_a	Mo K_a
Q_{min}/°	1.81	2.22	1.76
Q_{max}/°	30.51	36.10	32.40
Measured Refl's.	120931	33500	52819
Indep't Refl's	8676	12667	11167
Refl's I≥2 *s*(I)	8046	11824	7769
R_{int}	0.0427	0.0268	0.0605
Parameters	622	685	793
Restraints	0	1	0
Largest Peak	0.4222	0.2323	0.8004
Deepest Hole	-0.3613	-0.2021	-0.6920
GooF	1.1017	0.9885	1.0104
wR_2 (all data)	0.0326	0.0266	0.0436
wR_2	0.0318	0.0259	0.0372
R_1 (all data)	0.0252	0.0277	0.0781
R_1	0.0206	0.0234	0.0366

[a] - this structure contained one acetonitrile solvent molecule within the asymmetric unit.

Table S2. Important X-ray data collection parameters for the [Cu(^{R}**Bai**)PCy$_3$)] complexes, **4** - **6**.

**Compound**	**[Cu(^{Me}Bai)PCy$_3$] (4)** [a]	**[Cu(^{Me}Bai)PCy$_3$] (4)** [a]	**[Cu(^{Ph}Bai)PCy$_3$] (5)**	**[Cu(^{Naphth}Bai)PCy$_3$] (6)** [b]
CCDC No:	2263430	2263431	2263432	2263433
NCS No:	2019ncsx0403az	2019nsx0320y	2023ncs0072p	2022ncs0375z
Formula	$C_{33}H_{47}BCuN_4P$	$C_{33}H_{47}BCuN_4P$	$C_{38}H_{49}BCuN_4P$	$C_{44}H_{54}BCuN_5P$
$D_{calc.}$/g cm^{-3}	1.292	1.298	1.280	1.321
m/mm^{-1}	0.782	0.786	0.710	0.654
Formula Weight	605.06	605.06	667.183	758.296
Colour	colourless	colourless	colourless	colourless
Shape	block (cut)	block	block-shaped	block-shaped
Size/mm^3	0.07×0.06×0.05	0.06×0.05×0.04	0.11×0.08×0.06	0.10×0.09×0.06
T/K	100(2)	100(2)	100.00(10)	100(2)
Crystal System	triclinic	triclinic	monoclinic	monoclinic
Space Group	P-1	P-1	Cc	$P2_1/n$
a/Å	9.5265(2)	9.5075(2)	17.4362(3)	12.8295(2)
b/Å	10.1703(3)	10.1421(3)	11.11950(15)	16.0778(2)
c/Å	16.5786(5)	16.5905(4)	17.8532(3)	19.4995(2)
a/°	78.203(2)	78.149(2)	90	90
b/°	81.587(2)	81.522(2)	90.0101(13)	108.570(1)
g/°	88.993(2)	89.007(2)	90	90
V/Å^3	1555.28(7)	1548.41(7)	3461.42(9)	3812.75(9)
Z	2	2	4	4
Z'	1	1	1	1
Wavelength/Å	0.71075	0.71075	0.71075	0.71075
Radiation type	Mo K_a	Mo K_a	Mo K_a	Mo K_a
Q_{min}/°	2.046	2.052	2.17	2.10
Q_{max}/°	30.507	30.509	36.16	33.76
Measured Refl's.	28133	39865	71453	147120
Indep't Refl's	9476	9131	15267	14002
Refl's I≥2 s(I)	6984	7086	14540	11877
R_{int}	0.0585	0.0900	0.0469	0.0398
Parameters	474	474	582	955
Restraints	582	582	236	0
Largest Peak	0.511	0.831	1.1400	0.4508
Deepest Hole	-0.497	-0.415	-3.5224	-0.3402
GooF	1.021	1.065	1.0015	1.0736
wR_2 (all data)	0.1105	0.1157	0.1986	0.0345
wR_2	0.0981	0.1062	0.1969	0.0322
R_1 (all data)	0.0728	0.0866	0.0858	0.0315
R_1	0.0454	0.0566	0.0824	0.0204

[a] - two crystal structures were obtained for Complex **4**, 2019ncsx0403az and 2023ncs0072p. 2019ncsx0403az gave the higher quality solution and this was utilised for the structural analysis; [b] - this structure contained one acetonitrile solvent molecule within the asymmetric unit.

Table S3. Important X-ray data collection parameters for the [Cu(^{R}**Bai**)$_2$)] complexes, **7** and **8**.

**Compound**	**[Cu(^{Me}Bai)$_2$] (7)**	**[Cu(^{Ph}Bai)$_2$] (8)** [a]
CCDC No:	2263434	2263435
NCS No:	2019ncsx0403bz_twin1_hklf4	2021ncs0053z
Formula	$C_{30}H_{28}B_2CuN_8$	$C_{41}H_{34}B_2Cl_2CuN_8$
$D_{calc.}$/g cm^{-3}	1.487	1.462
m/mm^{-1}	0.873	0.797
Formula Weight	585.76	794.82
Colour	brown	light brown
Shape	prism	block (cut)
Size/mm^3	0.12×0.08×0.04	0.10×0.08×0.07
T/K	100(2)	100(2)
Crystal System	monoclinic	triclinic
Space Group	$P2_1/c$	*P*-1
a/Å	9.4133(3)	12.66630(10)
b/Å	16.7930(3)	12.84260(10)
c/Å	8.8715(2)	13.39790(10)
a/°	90	69.5070(10)
b/°	111.085(3)	62.6640(10)
g/°	90	85.2480(10)
V/Å^3	1308.49(6)	1805.54(3)
Z	2	2
Z'	0.5	1
Wavelength/Å	0.71075	0.71075
Radiation type	Mo K_a	Mo K_a
Q_{min}/°	2.319	1.700
Q_{max}/°	30.533	33.559
Measured Refl's.	8906	182758
Indep't Refl's	8906	13229
Refl's I≥2 *s*(I)	7016	11783
R_{int}	.	0.0475
Parameters	192	524
Restraints	0	17
Largest Peak	0.739	3.159
Deepest Hole	-0.551	-0.744
GooF	1.076	1.028
wR_2 (all data)	0.1355	0.1193
wR_2	0.1304	0.1152
R_1 (all data)	0.0614	0.0531
R_1	0.0477	0.0464

[a] - this structure contained one disordered DCM solvent molecule with two complexes within the asymmetric unit.

A2. Crystallographic data collection, reduction, and structure solution refinement.

CCDC numbers 2263427 - 2263435 contain supplementary crystallographic data for **[Cu(^{Me}Bai)PPh$_3$]**, **[Cu(^{Ph}Bai)PPh$_3$]**, **[Cu(^{Naphth}Bai)PPh$_3$]**, **[Cu(^{Me}Bai)PCy$_3$]**, **[Cu(^{Ph}Bai)PCy$_3$]**, **[Cu(^{Naphth}Bai)PCy$_3$]**, **[Cu(^{Me}Bai)$_2$]** and **[Cu(^{Ph}Bai)$_2$]**, respectively. These data can be obtained free of charge from the Cambridge crystallographic Data Centre via www.ccdc.cam.ac.uk/data_request/cif.

Details for [Cu(^{Me}Bai)PPh$_3$] (1) - 2019ncsx0289z

Single colourless prism-shaped crystals of **[Cu(^{Me}Bai)PPh$_3$]** were recrystallised from methanol by slow evaporation. A suitable crystal 0.20×0.14×0.09 mm^3 was selected and mounted on a MITIGEN holder in perfluoroether oil on a Rigaku FRE+ diffractometer with Arc)Sec VHF Varimax confocal mirrors, a UG2 goniometer and HyPix 6000HE detector. The crystal was kept at a steady T = 100(2) K during data collection. The structure was solved in the space group $P2_1/c$ (# 14) by using dual methods using the ShelXT 2018/2 [S1] structure solution program and refined by full matrix least squares minimisation on F^2 using olex2.refine 1.5-alpha[S2]. All non-hydrogen atoms were refined anisotropically.

Experimental Absorption Process Details: CrysAlisPro 1.171.40.58a[S3] using spherical harmonicas implemented in SCALE3 ABSPACK scaling algorithm.

_olex2_refine_details: Refinement using NoSpherA2, an implementation of NOn-SPHERical Atom-form-factors in Olex2.[S4] NoSpherA2 implementation of HAR makes use of tailor-made aspherical atomic form factors calculated on-the-fly from a Hirshfeld-partitioned electron density (ED) - not from spherical-atom form factors. The ED is calculated from a gaussian basis set single determinant SCF wavefunction - either Hartree-Fock or DFT using selected functionals - for a fragment of the crystal. This fragment can be embedded in an electrostatic crystal field by employing cluster charges or modelled using implicit solvation models, depending on the software used. The following options were used: SOFTWARE: ORCA 5.0, PARTITIONING: NoSpherA2, INT ACCURACY: Normal, METHOD: R2SCAN, BASIS SET: def2-TZVP, CHARGE: 0, MULTIPLICITY: 1, DATE: 2023-04-13_12-43-32

Details for [Cu(^{Ph}Bai)PPh$_3$] (2) - 2022ncs0565z

Single colourless rod-shaped crystals of **[Cu(^{Ph}Bai)PPh$_3$]** were recrystallised from hot MeCN. A suitable crystal 0.22×0.02×0.02 mm^3 was selected and mounted on a MITIGEN holder in oil on a Rigaku FRE+ diffractometer with Arc)Sec VHF Varimax confocal mirrors, a UG2 goniometer and HyPix 6000HE detector. The crystal was kept at a steady T = 100.00(10) K during data collection. The structure was solved in the space group $P2_1$ (# 4) by using dual methods using the ShelXT 2018/2 [S1] structure solution program and refined by full matrix least squares minimisation on $\boldsymbol{F^2}$ using olex2.refine 1.5-alpha.[S2] All non-hydrogen atoms were refined anisotropic Ally.

Experimental Absorption Process Details: CrysAlisPro 1.171.42.79a[S5] using spherical harmonics, implemented in SCALE3 ABSPACK scaling algorithm.

_olex2_refine_details: Refinement using NoSpherA2, an implementation of NOn-SPHERical Atom-form-factors in Olex2.[S4] NoSpherA2 implementation of HAR makes use of tailor-made aspherical atomic form factors calculated on-the-fly from a Hirshfeld-partitioned electron density (ED) - not from spherical-atom form factors. The ED is calculated from a gaussian basis set single determinant SCF wavefunction - either Hartree-Fock or DFT using selected functionals for a fragment of the crystal. This fragment can be embedded in an electrostatic crystal field by employing cluster charges or modelled using implicit solvation models, depending on the software used. The following options were used: SOFTWARE: ORCA 5.0, PARTITIONING: NoSpherA2, INT ACCURACY: Normal, METHOD: B3LYP, BASIS SET: jorge-TZP-DKH, CHARGE: 0, MULTIPLICITY: 1, RELATIVISTIC: DKH2, DATE: 2023-01-10_17-45-34

Details for [Cu(^{Naphth}Bai)PPh$_3$] (3) - 2022ncs0364

Single colourless needle-shaped-shaped crystals of **[Cu(^{Naphth}Bai)PPh$_3$]** were recrystallised from MeCN by slow evaporation. A suitable crystal 0.30×0.02×0.01 mm^3 was selected and mounted on a MITIGEN holder in oil on a Rigaku FRE+ diffractometer with Arc)Sec VHF Varimax confocal mirrors, a UG2 goniometer and HyPix 6000HE detector. The crystal was kept at a steady *T* = 100(2) K during data collection. The structure was solved in the space group $P2_1/c$ (# 14) by using dual methods using the ShelXT 2018/2[S1] structure solution program and refined by full matrix least squares minimisation on F^2 using version of olex2.refine 1.5-alpha.[S2] All non-hydrogen atoms were refined anisotropically.

Experimental Absorption Process Details: CrysAlisPro 1.171.42.64a[S5] using spherical harmonics, implemented in SCALE3 ABSPACK scaling algorithm.

_refine_special_details: Refinement using NoSpherA2, an implementation of NOn-SPHERical Atom-form-factors in Olex2.[S4] NoSpherA2 implementation of HAR makes use of tailor-made aspherical atomic form factors calculated on-the-fly from a Hirshfeld-partitioned electron density (ED) - not from spherical-atom form factors. The ED is calculated from a gaussian basis set single determinant SCF wavefunction - either Hartree-Fock or DFT using selected functionals - for a fragment of the crystal. This fragment can be embedded in an electrostatic crystal field by employing cluster charges or modelled using implicit solvation models, depending on the software used. The following options were used: SOFTWARE: ORCA 5.0, PARTITIONING: NoSpherA2, INT ACCURACY: Normal, METHOD: PBE, BASIS SET: def2-TZVP, CHARGE: 0, MULTIPLICITY: 1, DATE: 2022-08-12_18-56-36.

Details for [Cu(^{Me}Bai)PCy$_3$] (4) - 2019ncsx0403az

Single colourless (cut) block-shaped crystals of **[Cu(^{Me}Bai)PCy$_3$]** were supplied. A suitable crystal 0.07×0.06×0.05 mm^3 was selected and mounted on a MITIGEN holder in perfluoroether oil on a Rigaku FRE+ equipped with VHF Varimax confocal mirrors and an AFC12 goniometer and HyPix 6000 detector. The crystal was kept at a steady *T* = 100(2) K during data collection. The structure was solved in the space group *P*-1 (# 2) by using dual methods using the ShelXT 2018/2 [S1] structure solution program and refined by full matrix least squares minimisation on F^2 using version 2018/3 of ShelXL.[S1] All non-hydrogen atoms were refined anisotropically. Most hydrogen atom positions were calculated geometrically and refined using the riding model, but some hydrogen atoms were refined freely.

Experimental Absorption Process Details: CrysAlisPro 1.171.40.68a[S3] using spherical harmonics as implemented in SCALE3 ABSPACK scaling algorithm.

Refinement_special_details: Two of the cyclohexane rings were disordered (ca. 69:31). All disorder components were refined with thermal restraints and 1,2 and 1,3 equal distance geometrical restraints between equivalent atom pairs. The positions of the H-atom bonded to B1 was calculated geometrically and refined with the riding model. The constraint was then removed and its position allowed to freely refine.

Details for [Cu(^{Me}Bai)PCy$_3$] (4) - 2019ncsx0320y (the structure above gave a better solution and was therefore used for the structural analysis within the manuscript)

Single colourless block-shaped crystals of **[Cu(^{Me}Bai)PCy$_3$]** were recrystallised from methanol by slow evaporation. A suitable crystal 0.06×0.05×0.04 mm^3 was selected and mounted on a MITIGEN holder in perfluoroether oil on a Rigaku FRE+ equipped with VHF Varimax confocal mirrors and an AFC12 goniometer and HyPix 6000 detector. The crystal was kept at a steady T = 100(2) K during data collection. The structure was solved with the ShelXT [S1] structure solution program using the using dual methods solution method and by using Olex2 [S2] as the graphical interface. The model was refined with version 2018/3 of ShelXL[S6] using full matrix least squares minimisation on F^2 minimisation.

_refine_special_details: Two of the cyclohexane rings were disordered (ca. 68:32). All disorder components were refined with thermal restraints and 1,2 and 1,3 equal distance geometrical restraints between equivalent atoms. H1A bonded to B1 was located in the difference map and then refined with a riding model.

_exptl_absorpt_process_details: CrysAlisPro[S3] using spherical harmonics as implemented in SCALE3 ABSPACK.

Details for [Cu(^{Ph}Bai)PCy$_3$] (5) - 2023ncs0072p

Single colourless block-shaped crystals of **[Cu(^{Ph}Bai)PCy$_3$]** were recrystallised from MeCN by slow evaporation. A suitable crystal 0.11×0.08×0.06 mm^3 was selected and mounted on a MITIGEN holder in oil on a Rigaku FRE+ diffractometer with Arc)Sec VHF Varimax confocal mirrors, a UG2 goniometer and HyPix 6000HE detector. The crystal was kept at a steady T = 100.00(10) K during data collection. The structure was solved in the space group *Cc* (# 9) by using direct methods using the ShelXS[S1] structure solution program and refined by full matrix least squares minimisation on F^2 using version of olex2.refine 1.5-alpha.[S2] All non-hydrogen atoms were refined anisotropically. Most hydrogen atom positions were calculated geometrically and refined using the riding model, but some hydrogen atoms were refined freely.

Experimental Absorption Process Details: CrysAlisPro 1.171.42.90a[S7] (Rigaku Oxford Diffraction, 2023) using spherical harmonics, implemented in SCALE3 ABSPACK scaling algorithm.

Refinement_special_details: PCy$_3$ group is disordered over two positions ca. 50:50, Thermal restraints applied to all disorder components. The hydride H-atom was located in the difference map and refined with the riding model.

Details for [Cu(^{Naphth}Bai)PCy$_3$] (6) - 2022ncs0375z

Single colourless block-shaped crystals of **[Cu(^{Naphth}Bai)PCy$_3$]** were recrystallised from hot MeCN. A suitable crystal 0.10×0.09×0.06 mm^3 was selected and mounted on a MITIGEN holder in oil on a Rigaku FRE+ diffractometer with Arc)Sec VHF Varimax confocal mirrors, a UG2 goniometer and HyPix 6000HE detector. The crystal was kept at a steady T = 100(2) K during data collection. The structure was solved in the space group $P2_1/n$ (# 14) by using dual methods using the ShelXT 2018/2[S1] structure solution program and refined by full matrix least squares minimisation on F^2 using version of olex2.refine 1.5-alpha.[S2] All non-hydrogen atoms were refined anisotropically.

Experimental Absorption Process Details: CrysAlisPro 1.171.42.67a[S5] using spherical harmonics, implemented in SCALE3 ABSPACK scaling algorithm.

_olex2_refine_details: Refinement using NoSpherA2, an implementation of NOn-SPHERical Atom-form-factors in Olex2.[S4] NoSpherA2 implementation of HAR makes use of tailor-made aspherical atomic form factors calculated on-the-fly from a Hirshfeld-partitioned electron density (ED) - not from spherical-atom form factors. The ED is calculated from a gaussian basis set single determinant SCF wavefunction - either Hartree-Fock or DFT using selected functionals - for a fragment of the crystal. This fragment can be embedded in an electrostatic crystal field by employing cluster charges or modelled using implicit solvation models, depending on the software used. The following options were used: SOFTWARE: ORCA 5.0; PARTITIONING: NoSpherA2; INT ACCURACY: Normal; METHOD: B3LYP; BASIS SET: def2-SVP; CHARGE: 0; MULTIPLICITY: 1; DATE: 2023-01-15_16-31-07

Details for [Cu(^{Me}Bai)$_2$] (7) - 2019ncsx0403bz_twin1

Single brown prism-shaped crystals of **[Cu(^{Me}Bai)$_2$]** were supplied. A suitable crystal 0.12×0.08×0.04 mm^3 was selected and mounted on a MITIGEN holder in perfluoroether oil on a Rigaku FRE+ equipped with VHF Varimax confocal mirrors and an AFC12 goniometer and HyPix 6000 detector. The crystal was kept at a steady T = 100(2) K during data collection. The structure was solved in the space group $P2_1/c$ (# 14) by using dual methods using the ShelXT[S1] structure solution program and refined by full matrix least squares minimisation on F^2 using version 2018/3 of ShelXL.[S6] All non-hydrogen atoms were refined anisotropically. Most hydrogen atom positions were calculated geometrically and refined using the riding model, but some hydrogen atoms were refined freely.

Experimental Absorption Process Details: CrysAlisPro[S3] using spherical harmonics as implemented in SCALE3 ABSPACK scaling algorithm.

Refinement_special_details: The position of the H-atom bonded to B1 was calculated geometrically and refined with the riding model before the constraint was then removed and its position allowed to freely refine.

Twin_special_details: Component 2 rotated by 3.5523° around [0.08 -0.98 0.17] (reciprocal) or [0.39 -0.71 58] (direct)

Details for [Cu(^{Ph}Bai)$_2$] (8) - 2021ncs0053z

Single light brown block crystals of **[Cu(^{Ph}Bai)$_2$]** recrystallised from a mixture of DCM and hexane by slow evaporation. A suitable crystal with dimensions 0.10 × 0.08 × 0.07 mm^3 was selected and mounted on a MITIGEN holder in perfluoroether oil on a Rigaku FRE+ equipped with VHF Varimax confocal mirrors and an AFC12 goniometer and HyPix 6000 detector diffractometer. The crystal was kept at a steady *T* = 100(2) K during data collection. The structure was solved and the space group *P*-1 (# 2) determined by the ShelXT 2018/2[S1] structure solution program using dual methods and refined by full matrix least squares minimisation on F^2 using ShelXL 2018/3.[S6] All non-hydrogen atoms were refined anisotropically. Hydrogen atom positions were calculated geometrically and refined using the riding model. Most hydrogen atom positions were calculated geometrically and refined using the riding model, but some hydrogen atoms were refined freely (see below).

Experimental Absorption Process Details: CrysAlisPro[S8] using spherical harmonics as implemented in SCALE3 ABSPACK scaling algorithm.

Refinement_special_details: Solvent DCM disordered ca. 80:20. Geometric and thermal restraints applied to both disorder components. H-atoms bonded to B-atoms were refined with the riding model and then the geometrical restraints were removed, and their positions were allowed to freely refine.

Crystallography References

S1. Sheldrick, G.M., ShelXT-Integrated space-group and crystal-structure determination, *Acta Cryst.*, (2015), **A71**, 3-8.

S2. L.J. Bourhis and O.V. Dolomanov and R.J. Gildea and J.A.K. Howard and H. Puschmann, The Anatomy of a Comprehensive Constrained, Restrained, Refinement Program for the Modern Computing Environment - Olex2 Disected, *Acta Cryst. A*, (2015), **A71**, 59-71.

S3. CrysAlisPro Software System, Rigaku Oxford Diffraction, Poland (Rigaku, V1.171.40.67a, 2019).

S4. Florian Kleemiss, Oleg V. Dolomanov, Michael Bodensteiner, Norbert Peyerimhoff, Laura Midgley, Luc J. Bourhis, Alessandro Genoni, Lorraine A. Malaspina, Dylan Jayatilaka, John L. Spencer, Fraser White, Bernhard Grundkötter-Stock, Simon Steinhauer, Dieter Lentz, Horst Puschmann, and Simon Grabowsky, Chem. Sci., 2021,12, 1675-1692

S5. CrysAlisPro (Rigaku OD, Poland, V1.171.42.79a, 2022)

S6. Sheldrick, G.M., Crystal structure refinement with ShelXL, Acta Cryst., (2015), C27, 3-8.

S7. CrysAlisPro Software System, Rigaku Oxford Diffraction, Poland (2023).

S8. CrysAlisPro Software System, Rigaku Oxford Diffraction, (Rigaku, V1.171.41.103a, 2021).

B1 Spectra for [Cu{κ^3-*N,N,H*-^{Me}Bai}(PPh$_3$)] (1)

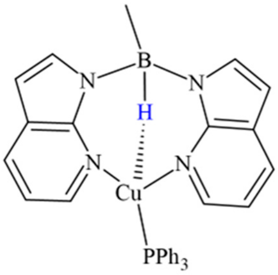

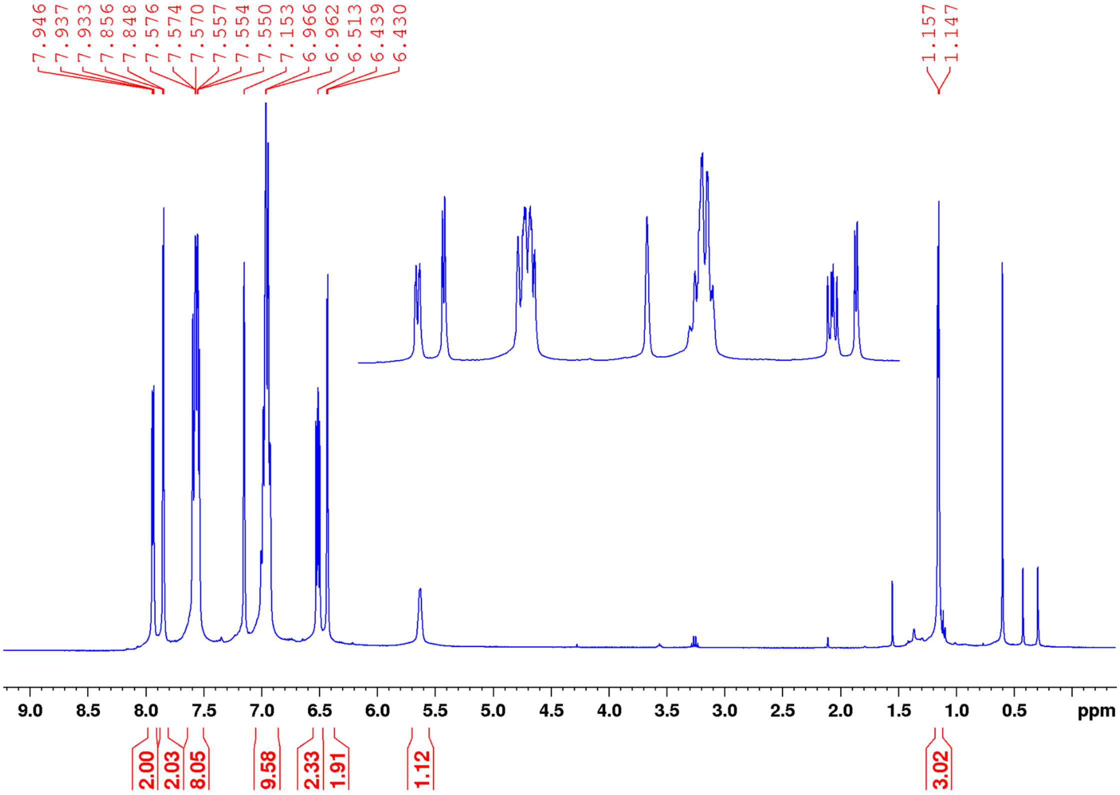

Fig S1.1: $^1H\{^{11}B\}$ NMR spectrum of the complex **[Cu{κ^3-*N,N,H*-^{Me}Bai}(PPh$_3$)]** C_6D_6 at 298 K.

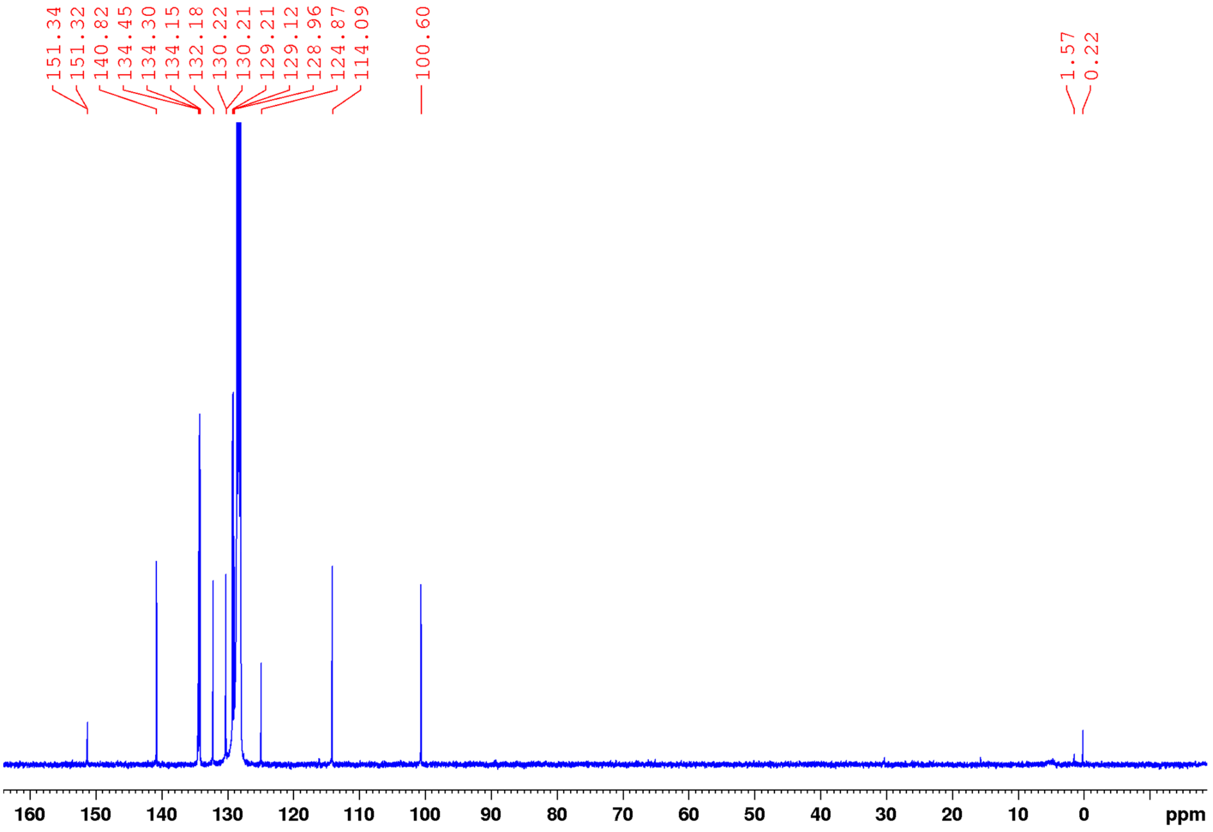

Fig S1.2: ^{13}C NMR spectrum of the complex **[Cu{κ³-*N,N,H*-^{Me}Bai}(PPh$_3$)]** C_6D_6 at 298 K.

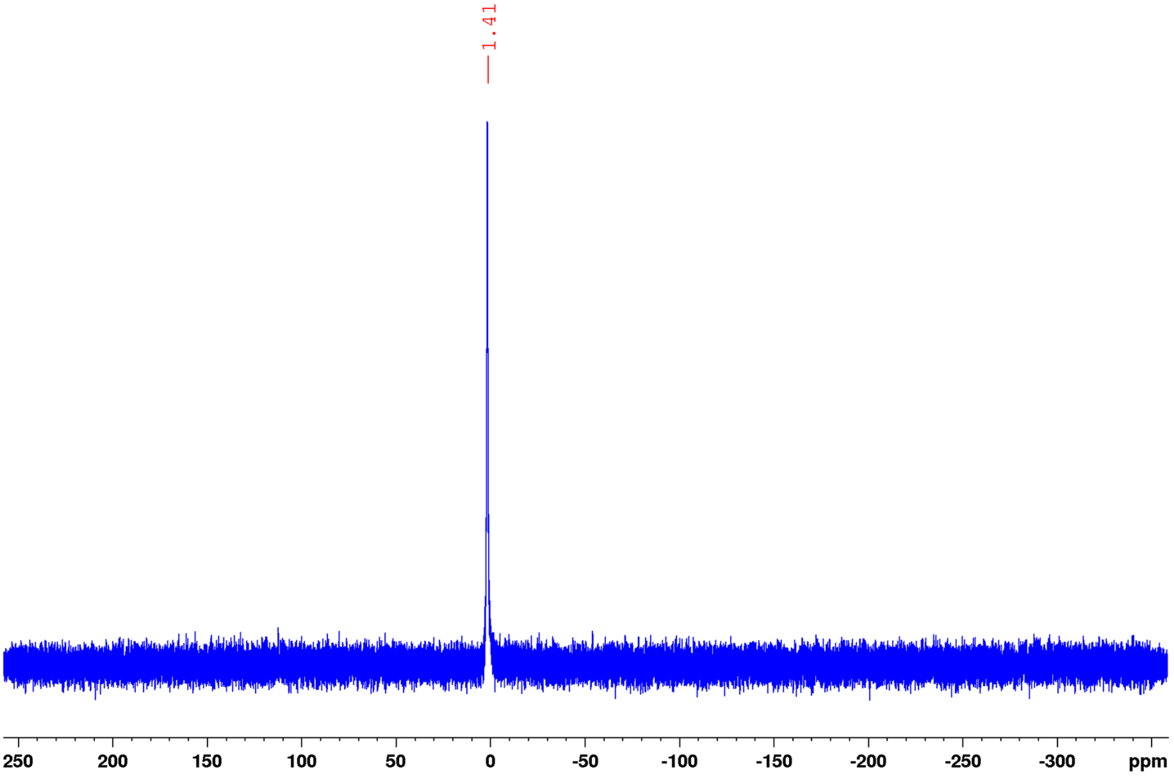

Fig S1.3: ^{31}P{^{1}H} NMR spectrum of the complex **[Cu{κ³-*N,N,H*-^{Me}Bai}(PPh$_3$)]** C_6D_6 at 298 K.

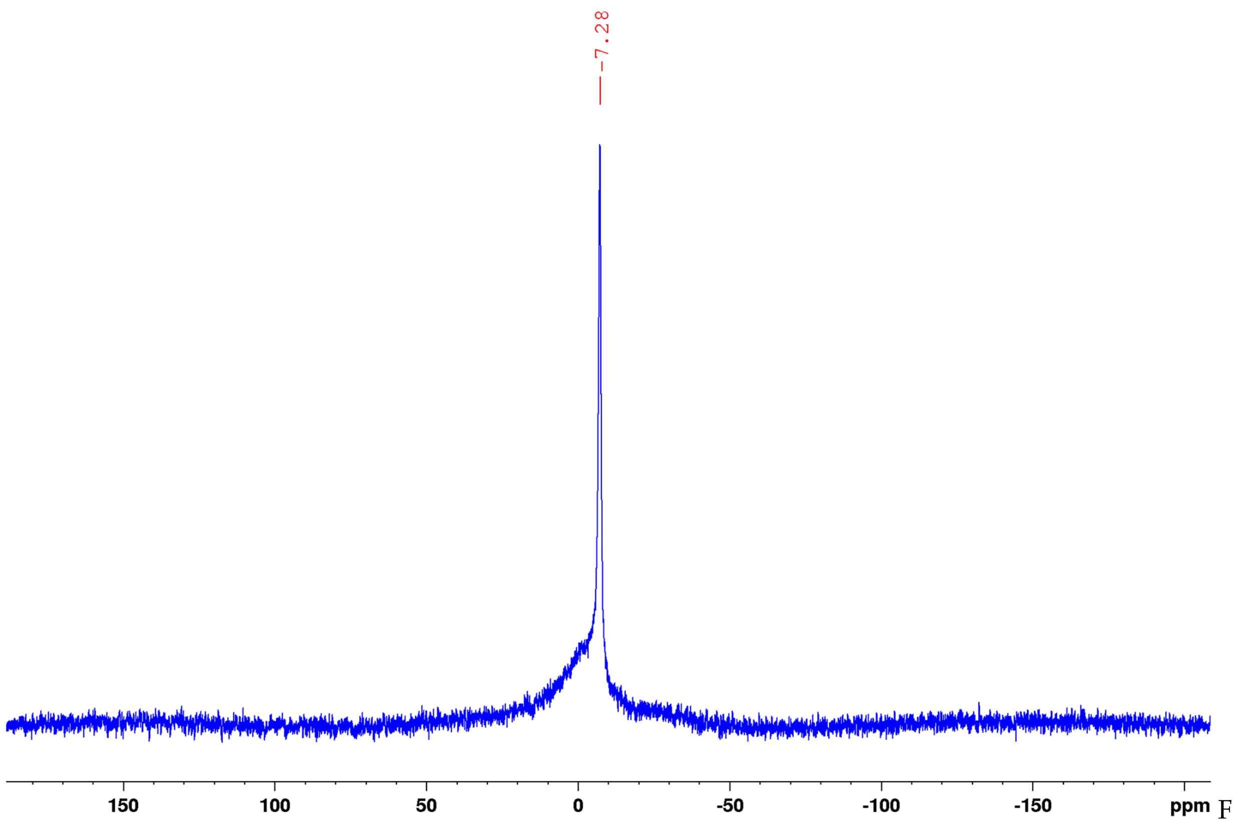

Fig S1.4: $^{11}B\{^{1}H\}$ NMR spectrum of the complex **[Cu{κ^3-*N,N,H*-^{Me}Bai}(PPh$_3$)]** C_6D_6 at 298 K.

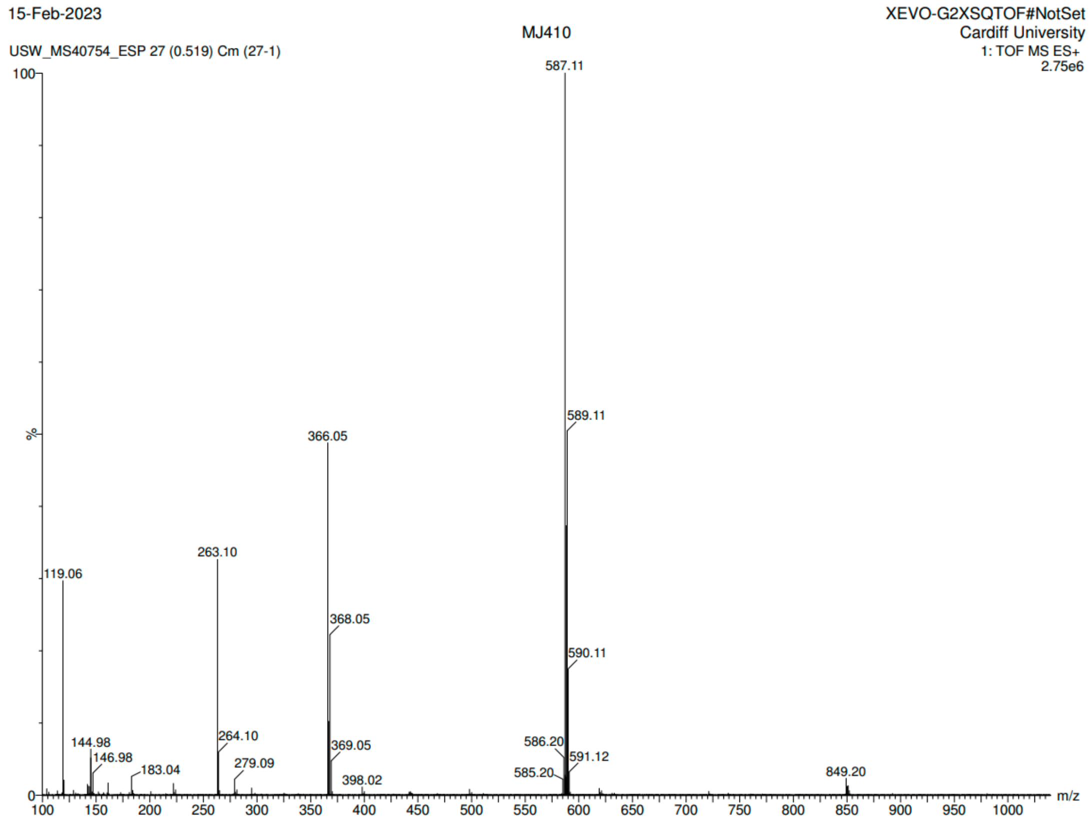

Fig S1.5: Mass spectrometry data for **[Cu{κ^3-*N,N,H*-^{Me}Bai}(PPh$_3$)]**

Synthesis of [Cu{κ³-N,N,H-^{Ph}Bai}(PPh_3)] (2)

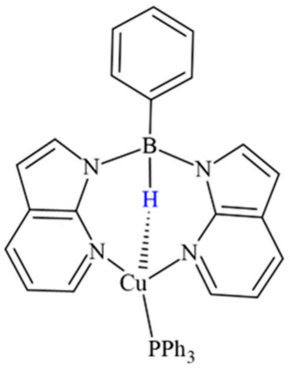

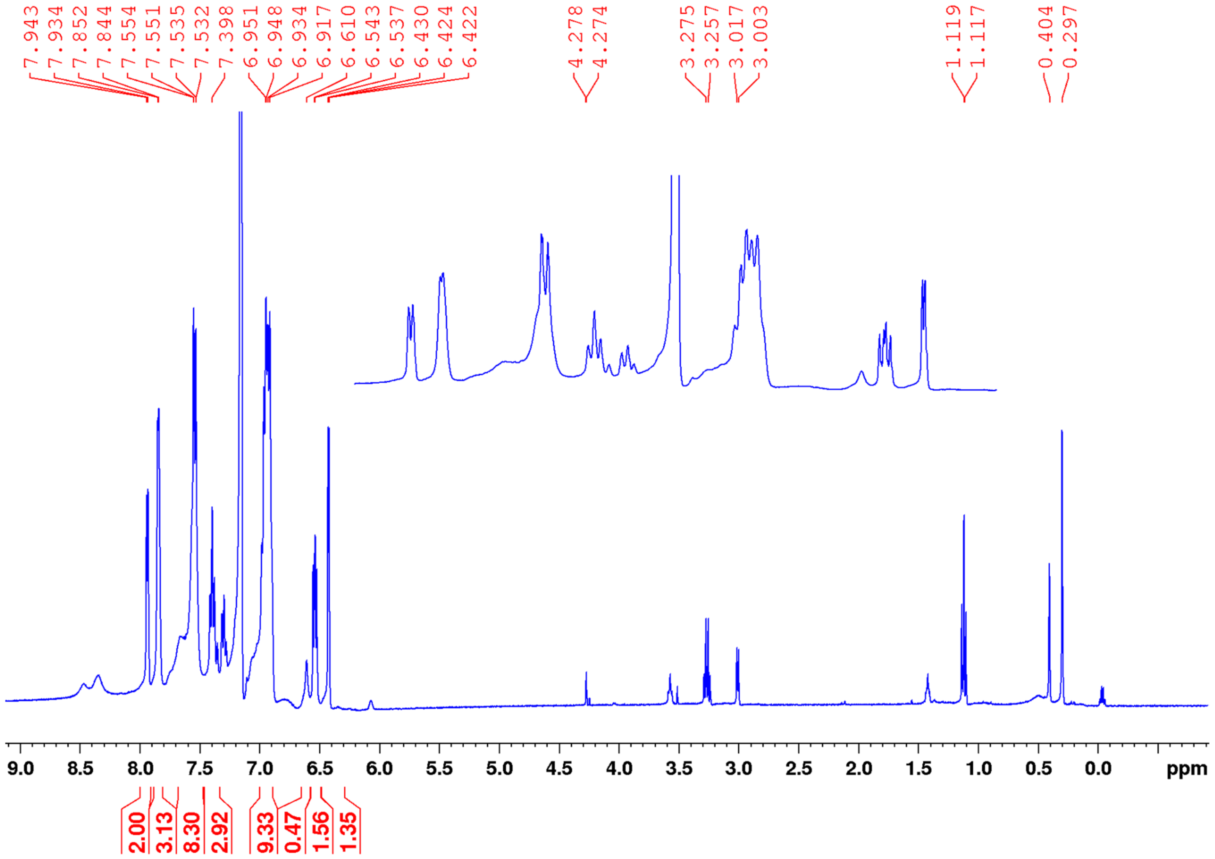

Fig S2.1: $^1H\{^{11}B\}$ NMR spectrum of the complex **[Cu{κ³-*N,N,H*-^{Ph}Bai}(PPh_3)]** C_6D_6 at 298 K.

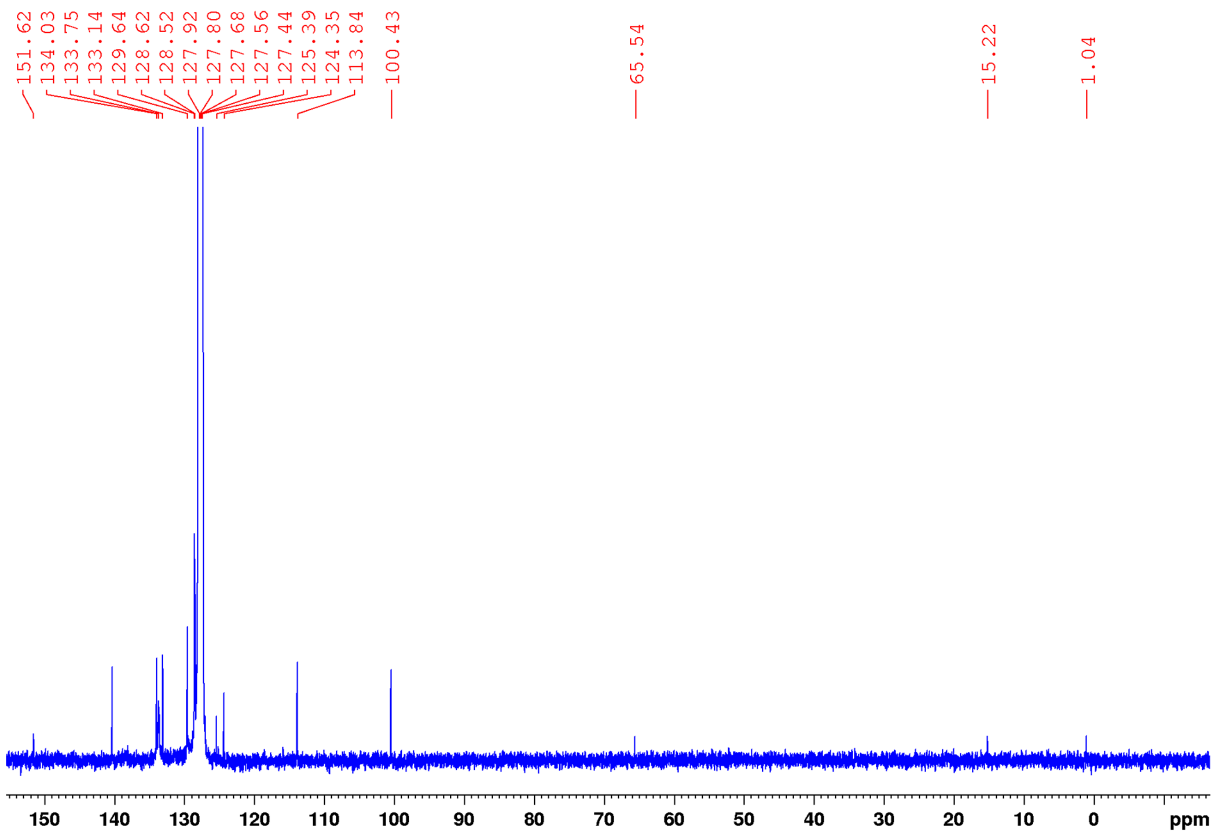

Fig S2.2: ^{13}C NMR spectrum of the complex **[Cu{κ[3]-*N,N,H*-[Ph]Bai}(PPh$_3$)]** C_6D_6 at 298 K.

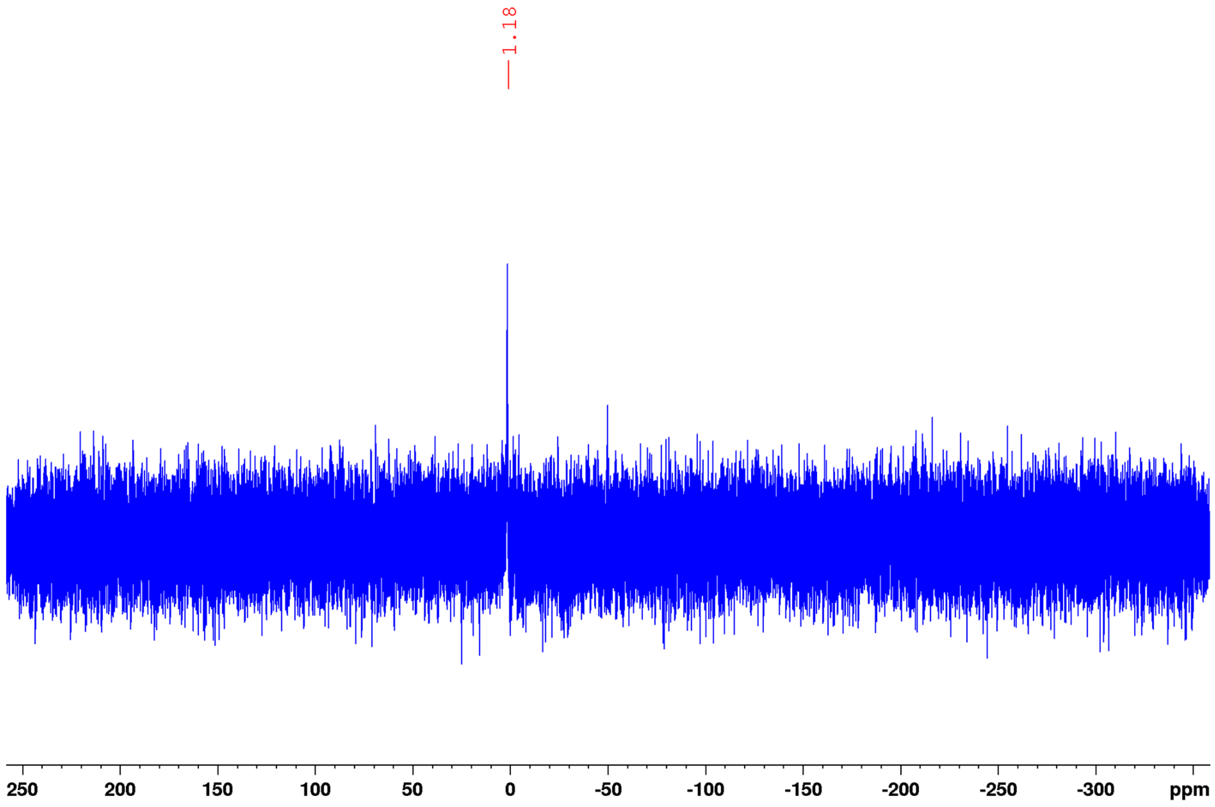

Fig S2.3: ^{31}P{^{1}H} NMR spectrum of the complex **[Cu{κ[3]-*N,N,H*-[Ph]Bai}(PPh$_3$)]** C_6D_6 at 298 K.

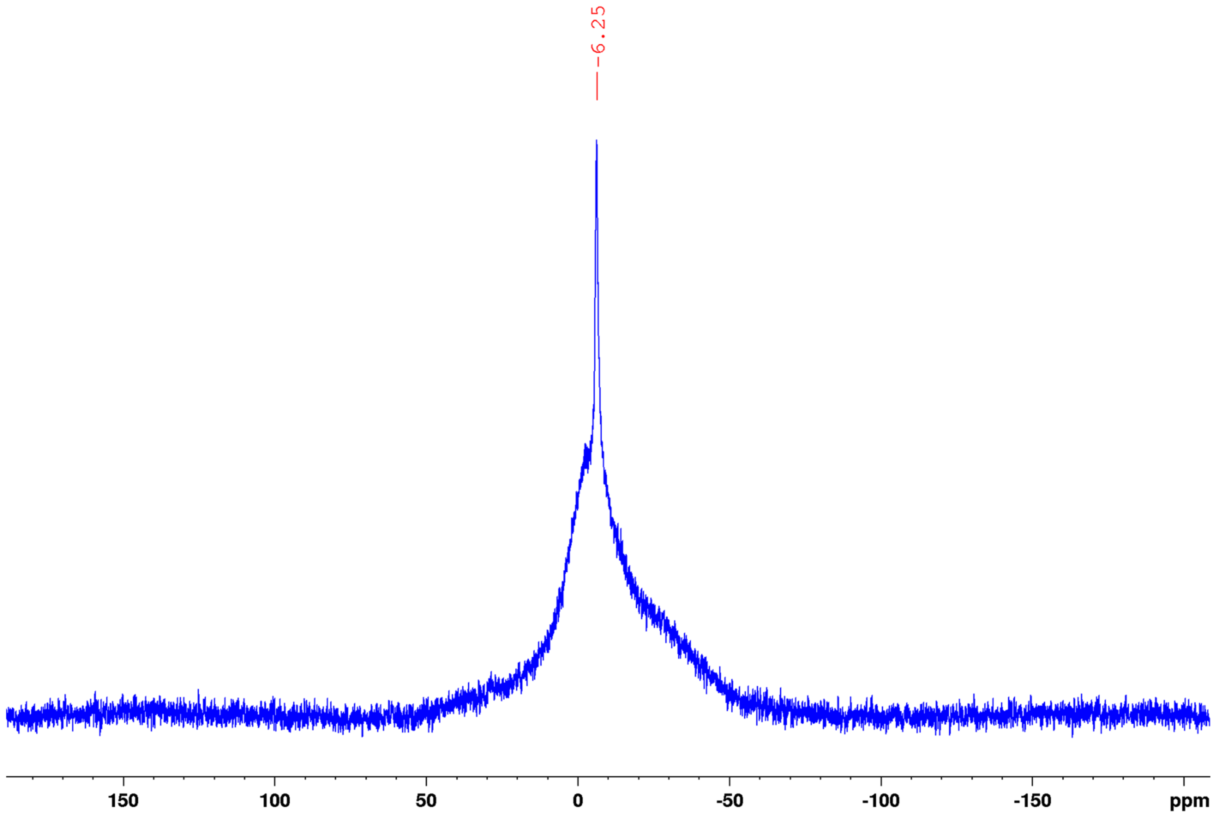

Fig S2.4: $^{11}B\{^{1}H\}$ NMR spectrum of the complex **[Cu{κ³-*N,N,H*-^{Ph}Bai}(PPh$_3$)]** C_6D_6 at 298 K.

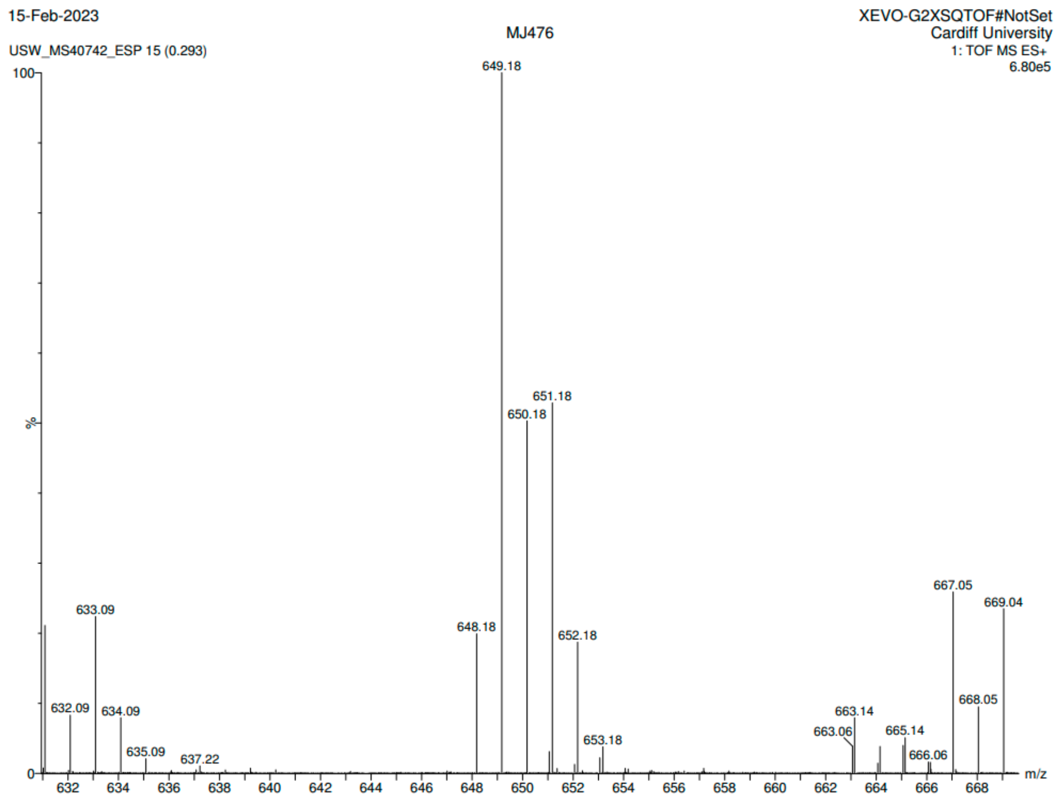

Fig S2.5: Mass spectrometry data for **[Cu{κ³-*N,N,H*-^{Ph}Bai}(PPh$_3$)]**

B3 Spectra for [Cu{κ3-*N,N,H*-NaphthBai}(PPh_3)] (3)

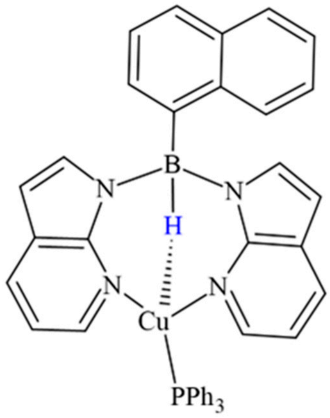

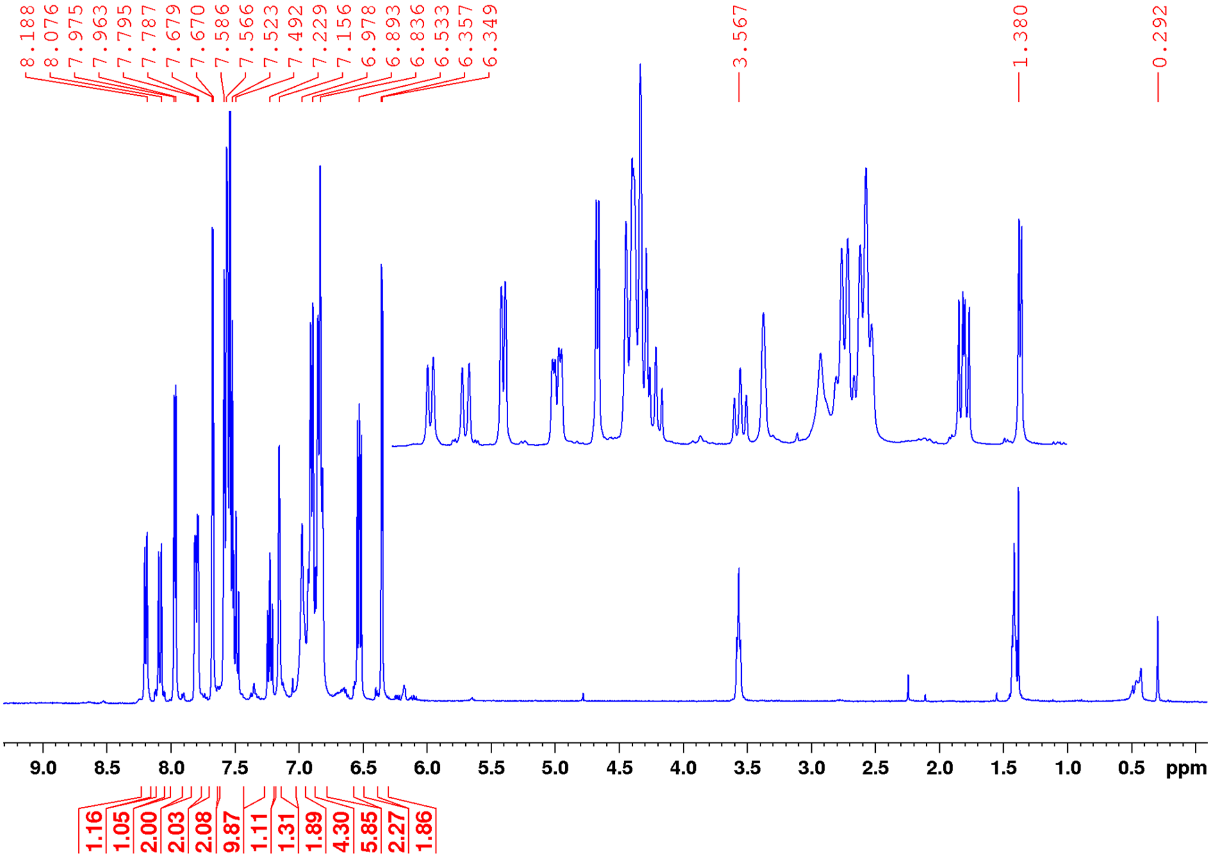

Fig S3.1: $^1H\{^{11}B\}$ NMR spectrum of the complex **[Cu{κ3-*N,N,H*-NaphthBai}(PPh_3)]** C_6D_6 at 298 K.

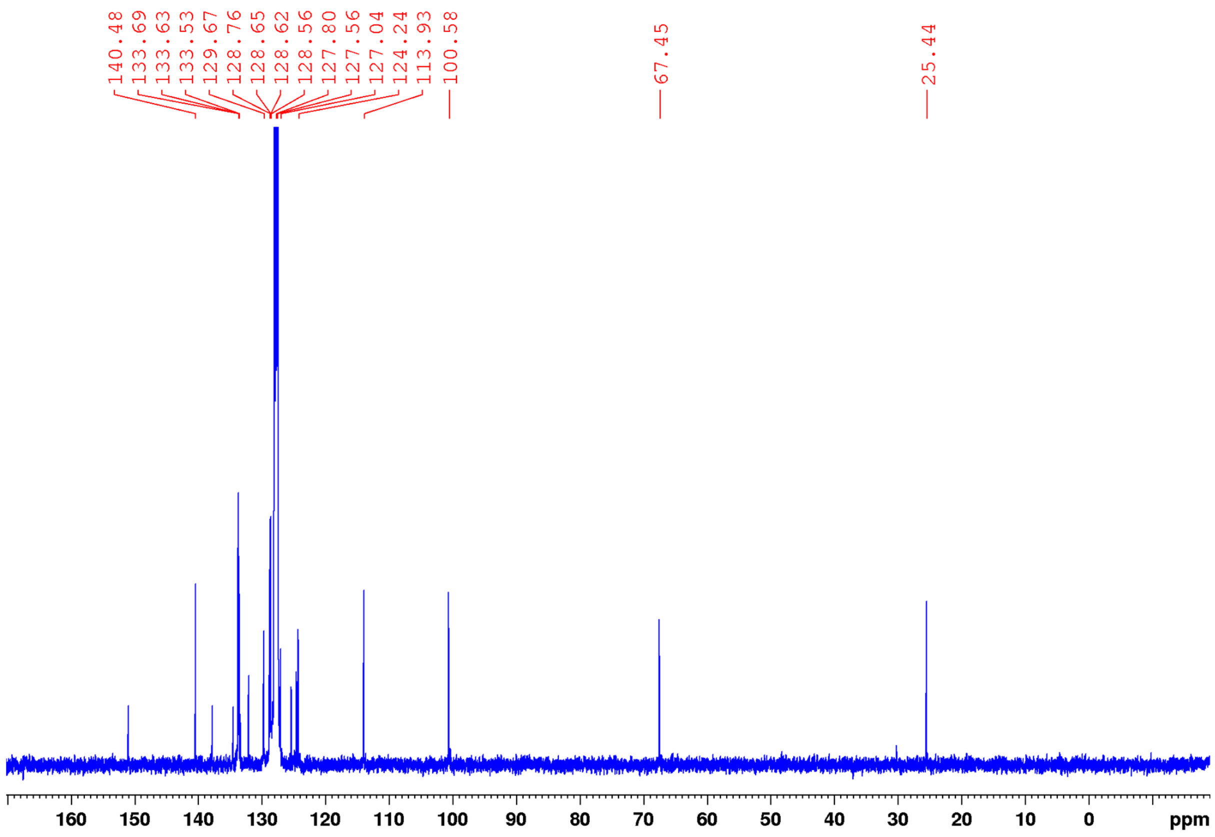

Fig S3.2: ^{13}C NMR spectrum of the complex **[Cu{κ^3-*N,N,H*-^{Naphth}Bai}(PPh$_3$)]** C_6D_6 at 298 K.

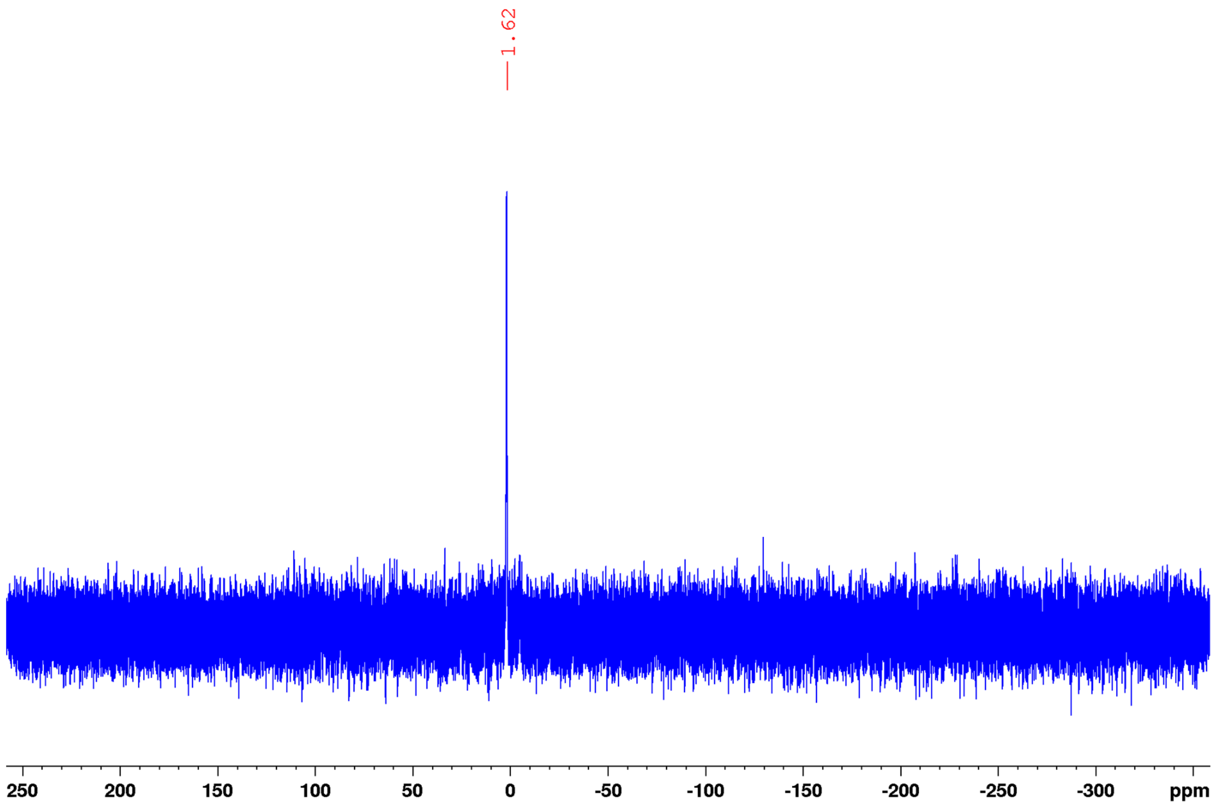

Fig S3.3: $^{31}P\{^1H\}$ NMR spectrum of the complex **[Cu{κ^3-*N,N,H*-^{Naphth}Bai}(PPh$_3$)]** C_6D_6 at 298 K.

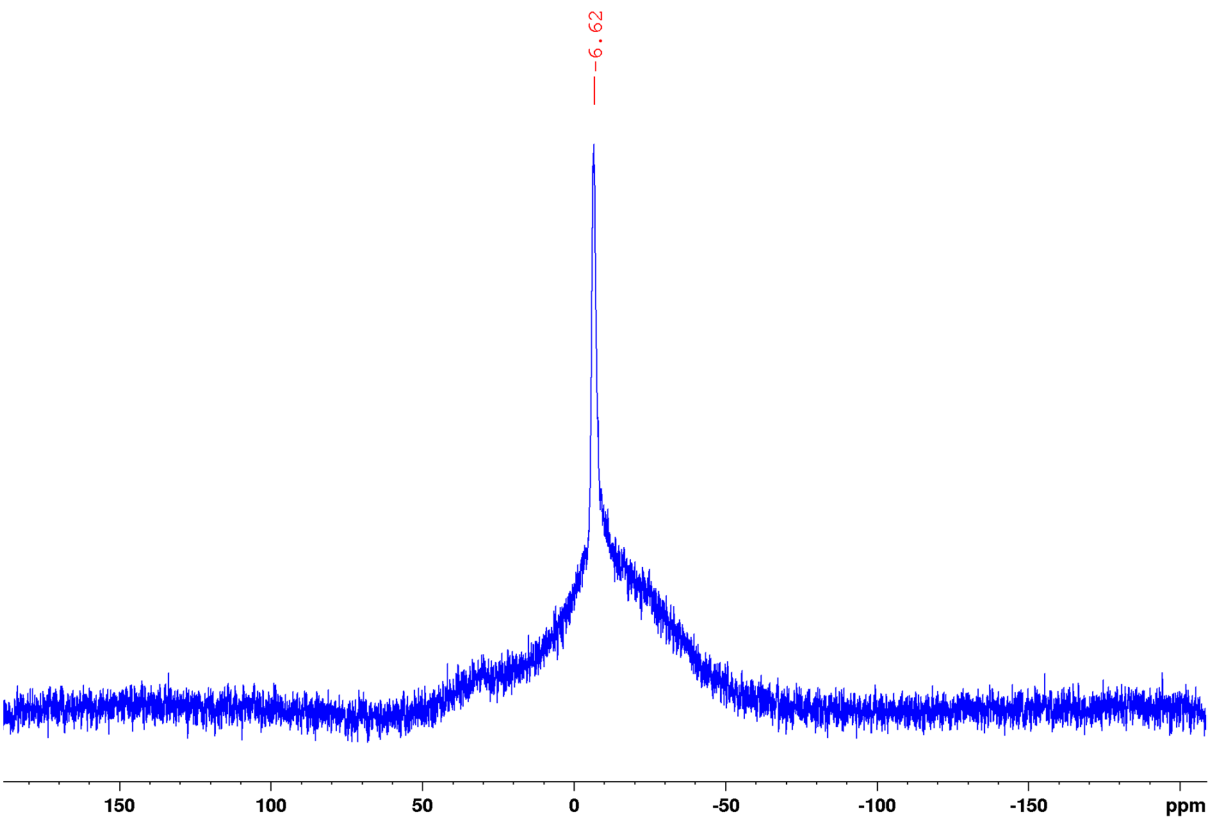

Fig S3.4: $^{11}B\{^{1}H\}$ NMR spectrum of the complex **[Cu{κ^3-*N,N,H*-^{Naphth}Bai}(PPh$_3$)]** C_6D_6 at 298 K.

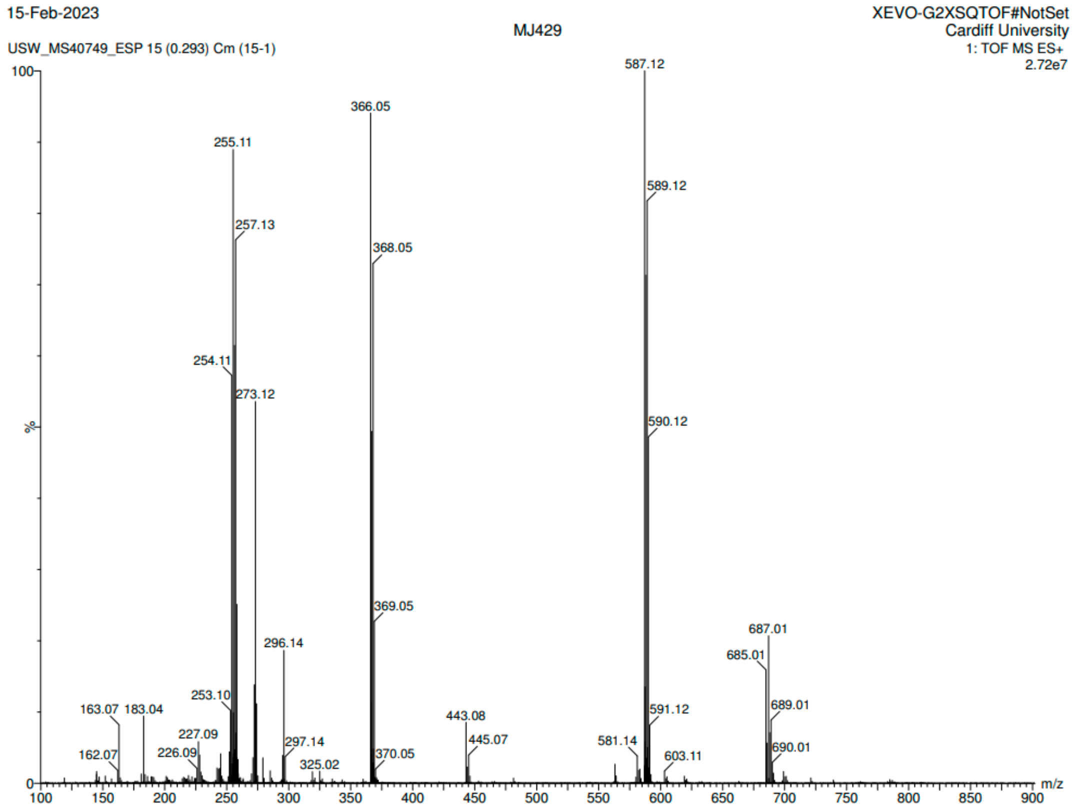

Fig S3.5: Mass spectrometry data for **[Cu{κ^3-*N,N,H*-^{Naphth}Bai}(PPh$_3$)]**

B4 Spectra for [Cu{κ^3-*N,N,H*-^{Me}Bai}(PCy_3)] (4)

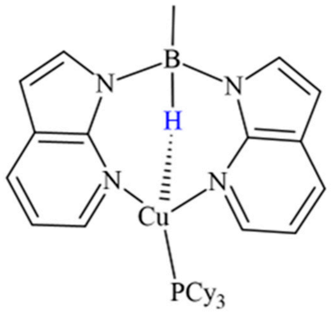

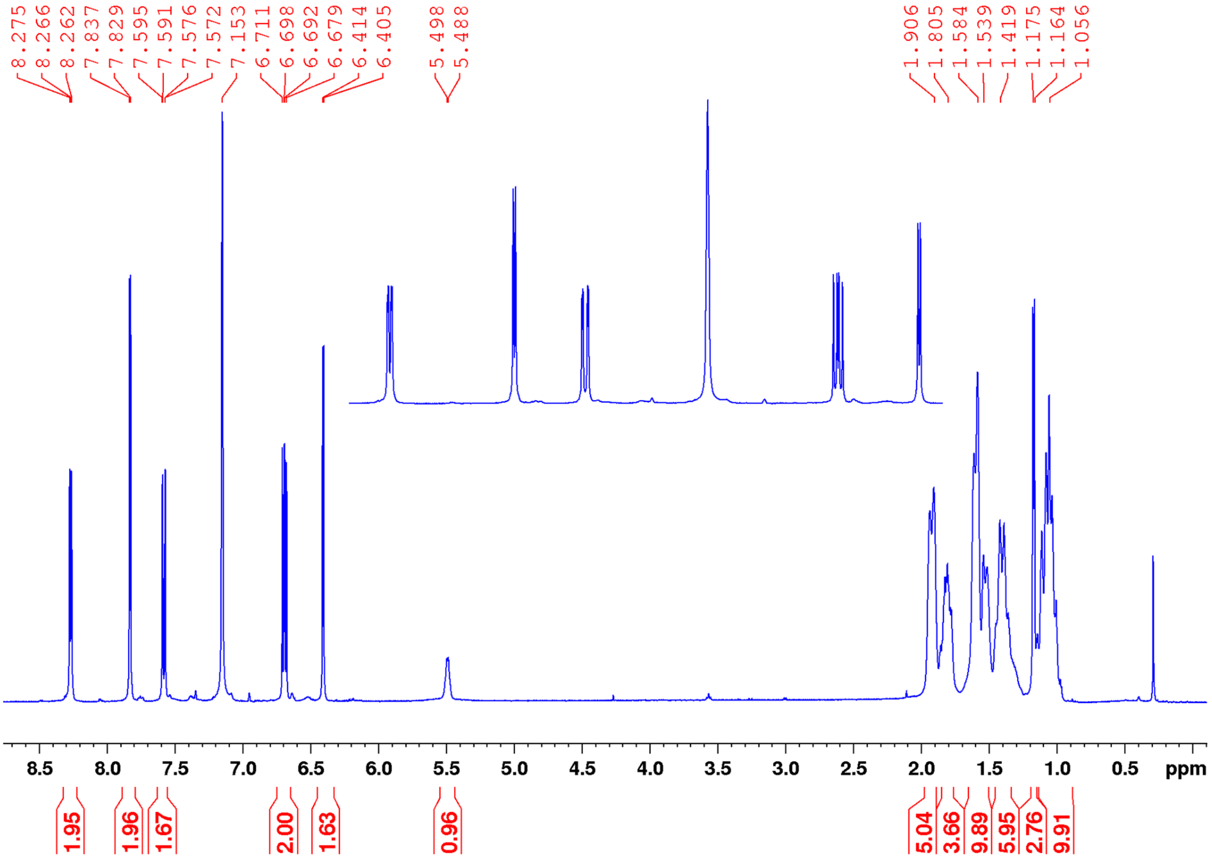

Fig S4.1: ^{1}H{^{11}B} NMR spectrum of the complex **[Cu{κ^3-*N,N,H*-^{Me}Bai}(PCy_3)]** C_6D_6 at 298 K.

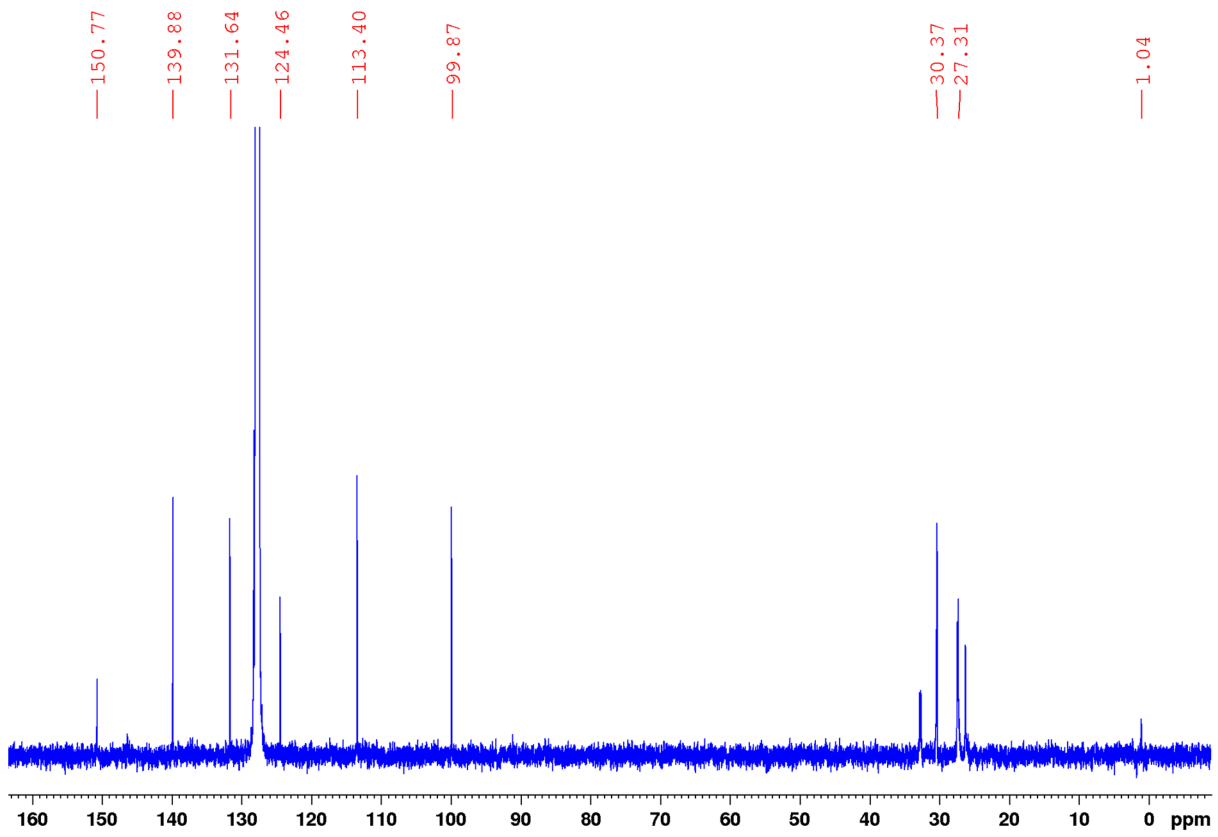

Fig S4.2: ^{13}C NMR spectrum of the complex **[Cu{κ³-*N,N,H*-^{Me}Bai}(PCy₃)]** C_6D_6 at 298 K.

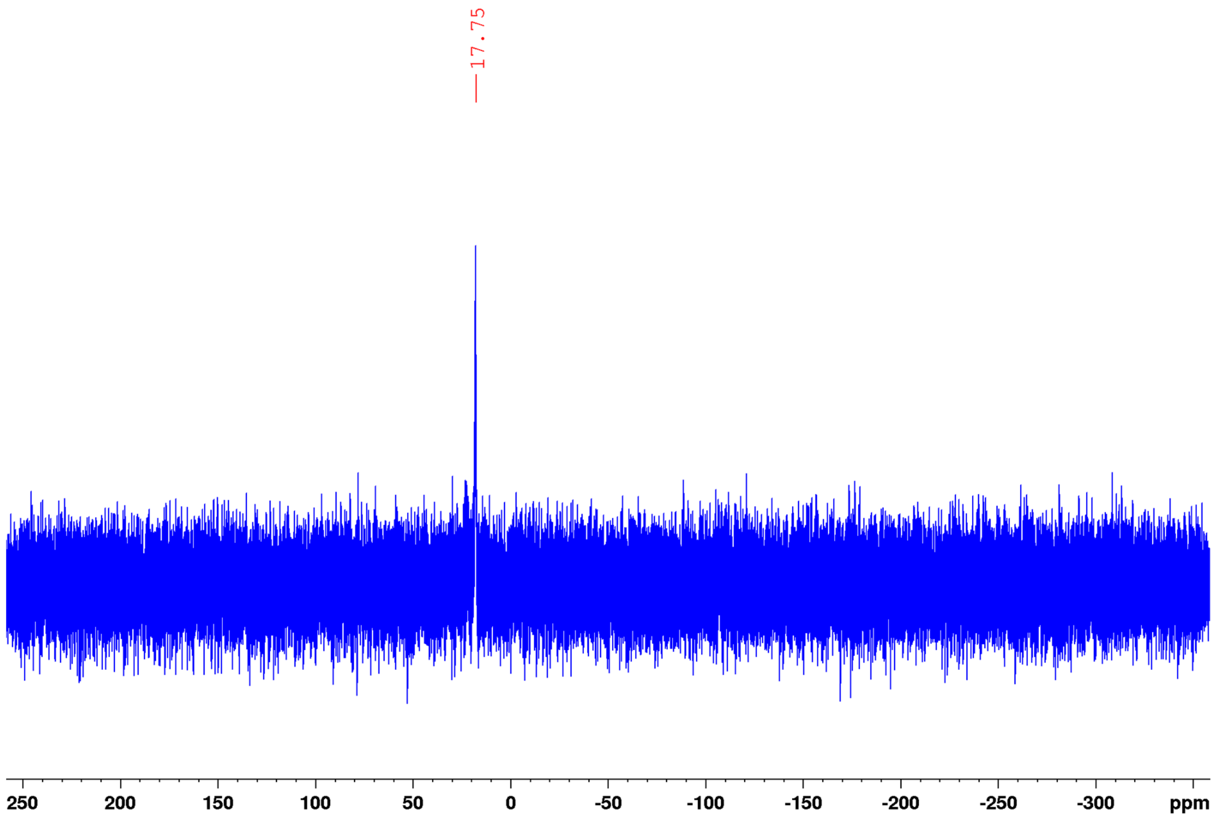

Fig S4.3: $^{31}P\{^1H\}$ NMR spectrum of the complex **[Cu{κ³-*N,N,H*-^{Me}Bai}(PCy$_3$)]** C_6D_6 at 298 K.

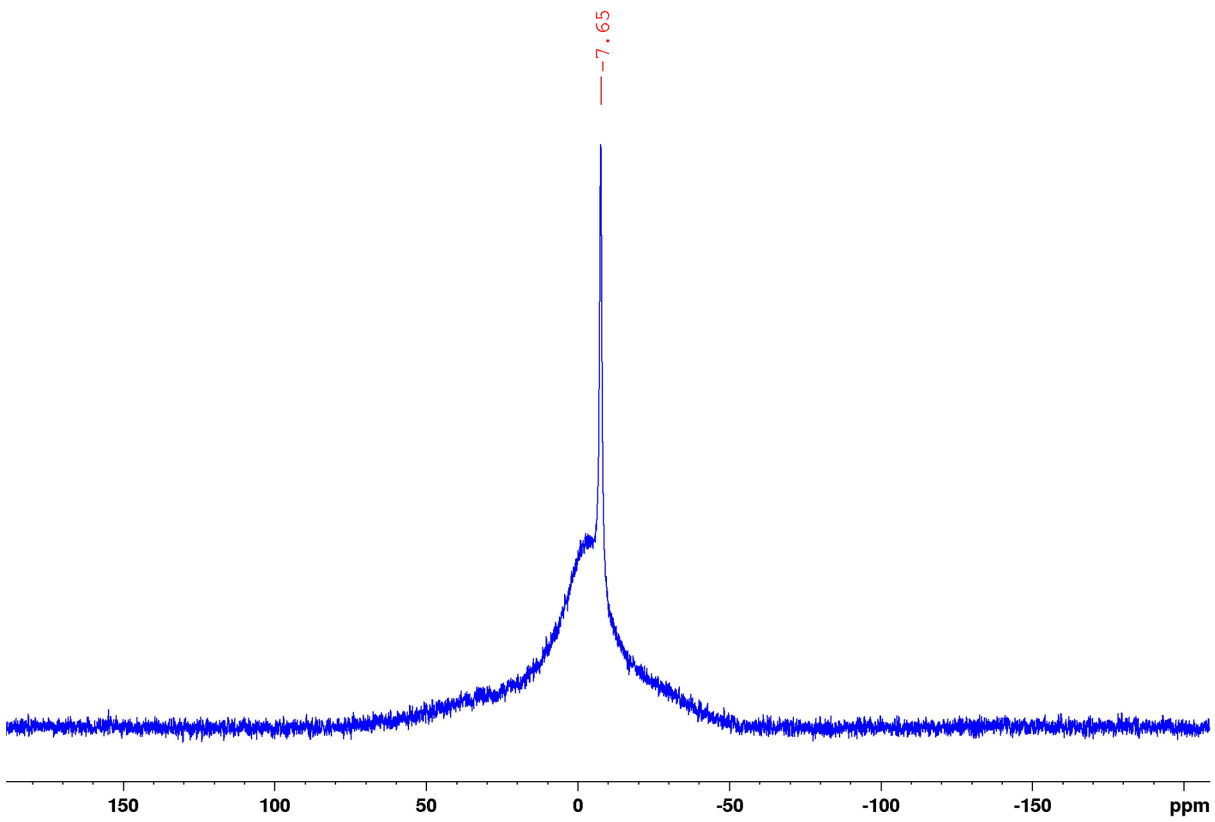

Fig S4.4: $^{11}B\{^{1}H\}$ NMR spectrum of the complex **[Cu{κ^3-*N,N,H*-^{Me}Bai}(PCy$_3$)]** C_6D_6 at 298 K.

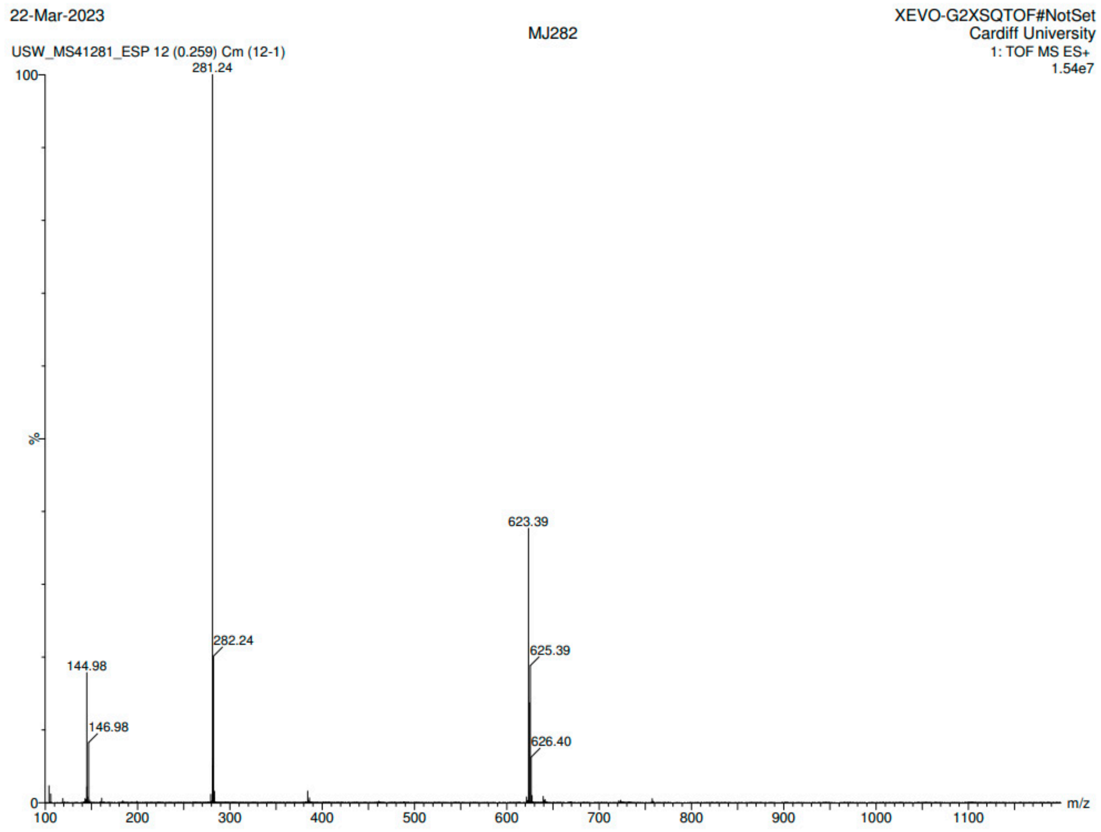

Fig 4.5: Mass spectrometry data for **[Cu{κ^3-*N,N,H*-^{Me}Bai}(PCy$_3$)]**

B5 Spectra for [Cu{κ³-*N,N,H*-^Ph^Bai}(PCy₃)] (5)

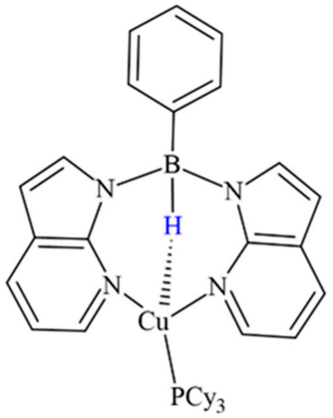

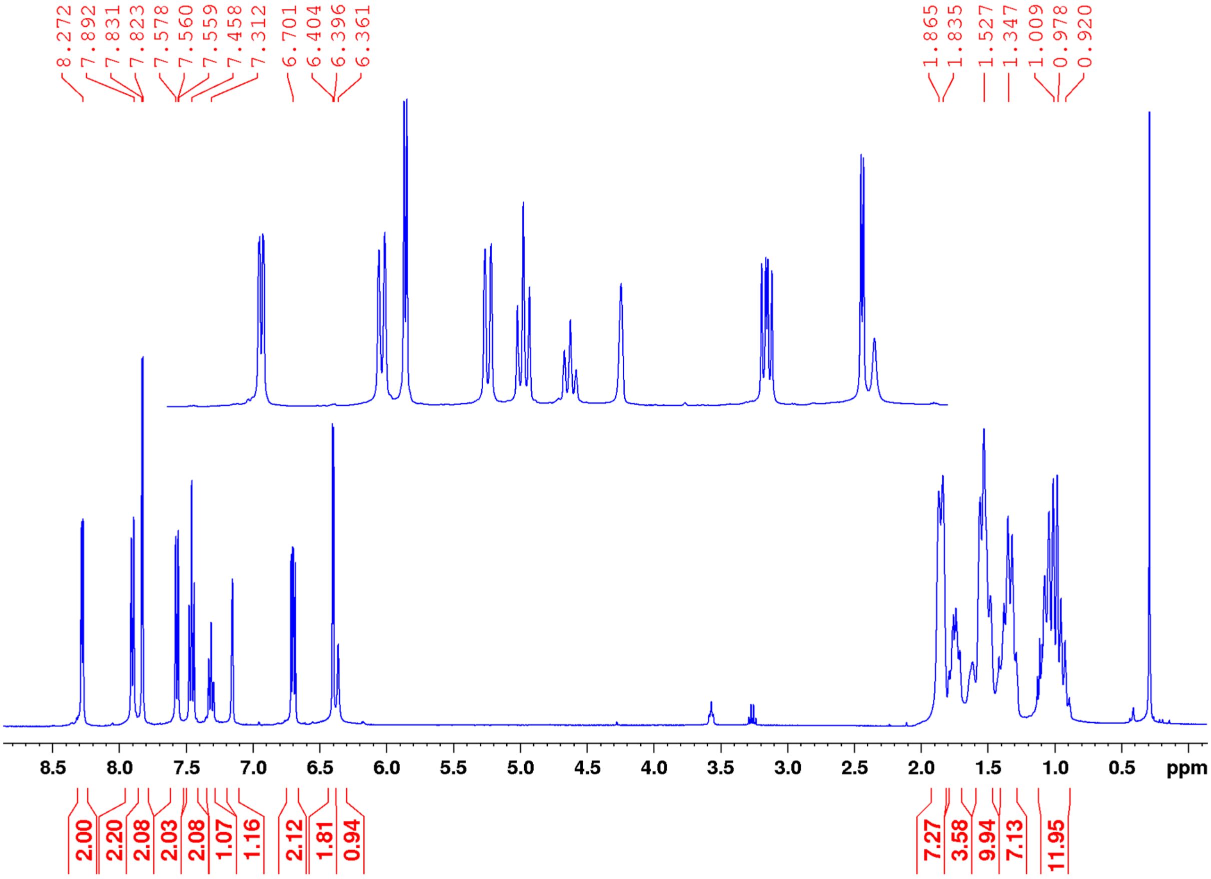

Fig S5.1: $^{1}H\{^{11}B\}$ NMR spectrum of the complex **[Cu{κ³-*N,N,H*-^Ph^Bai}(PCy₃)]** C_6D_6 at 298 K.

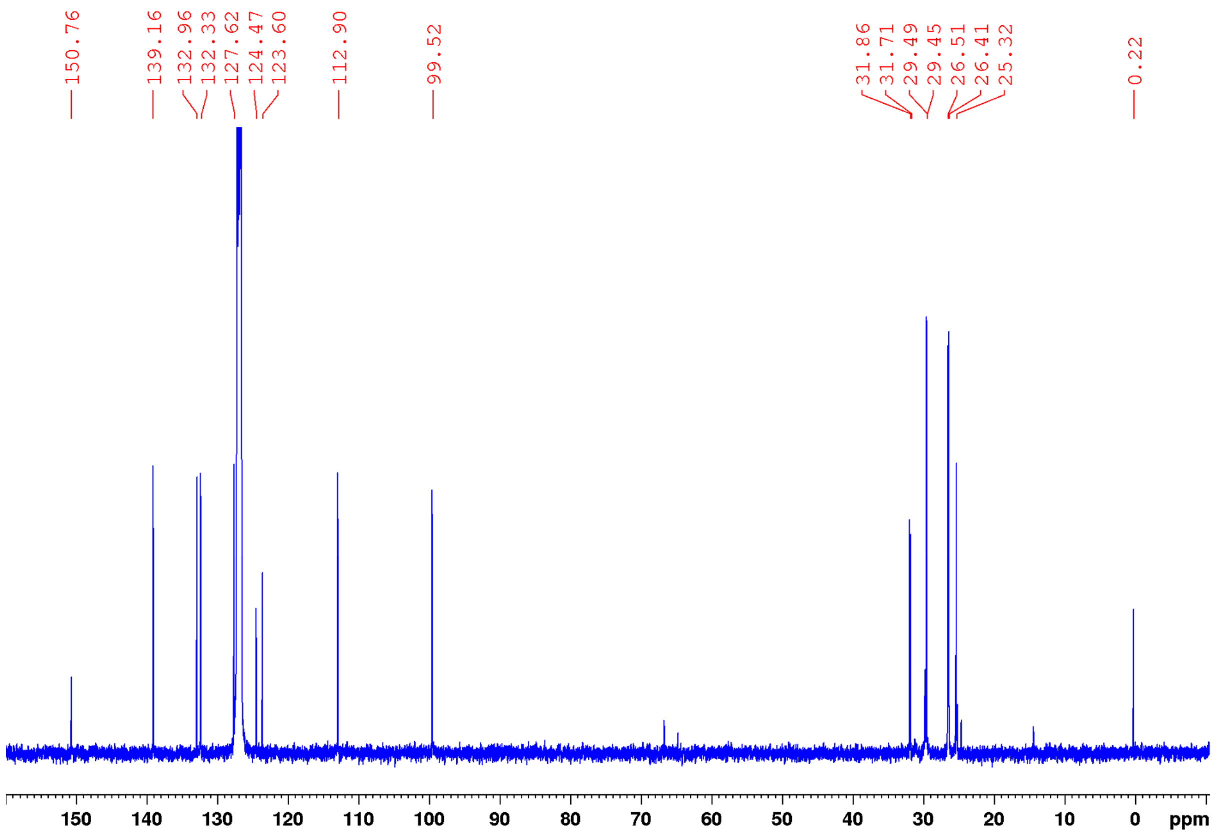

Fig S5.2: ^{13}C NMR spectrum of the complex **[Cu{κ^3-*N,N,H*-^{Ph}Bai}(PCy$_3$)]** C_6D_6 at 298K.

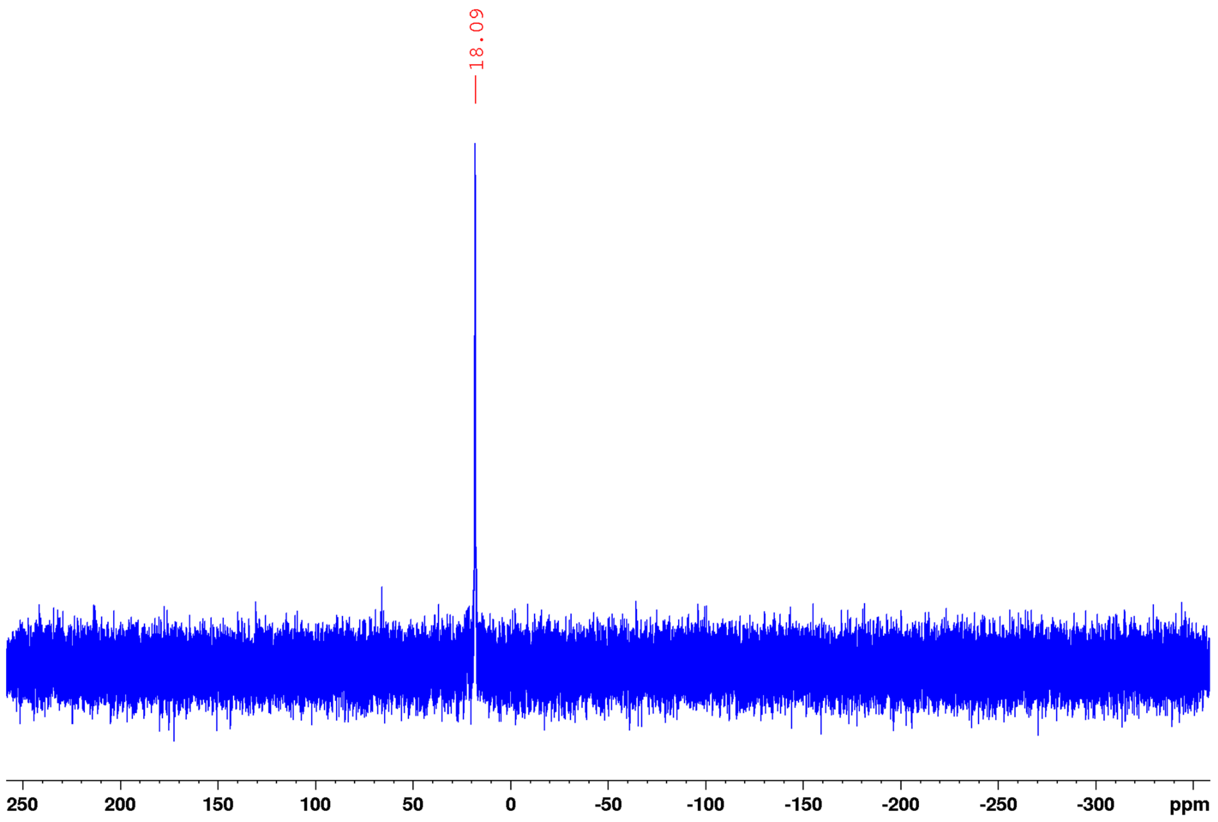

Fig S5.3: ^{31}P{^{1}H} NMR spectrum of the complex **[Cu{κ^3-*N,N,H*-^{Ph}Bai}(PCy$_3$)]** C_6D_6 at 298 K.

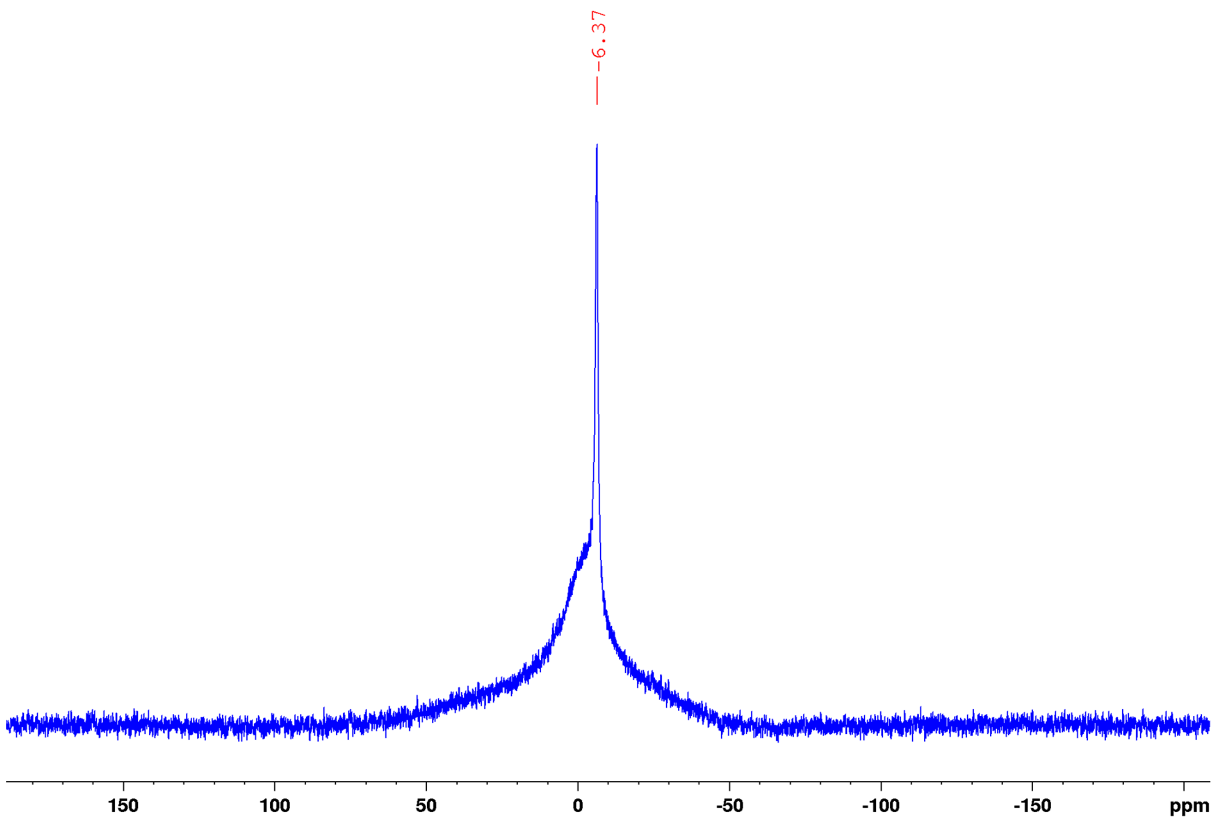

Fig S5.4: $^{11}B\{^{1}H\}$ NMR spectrum of the complex **[Cu{κ^3-*N,N,H*-^{Ph}Bai}(PCy$_3$)]** C_6D_6 at 298 K.

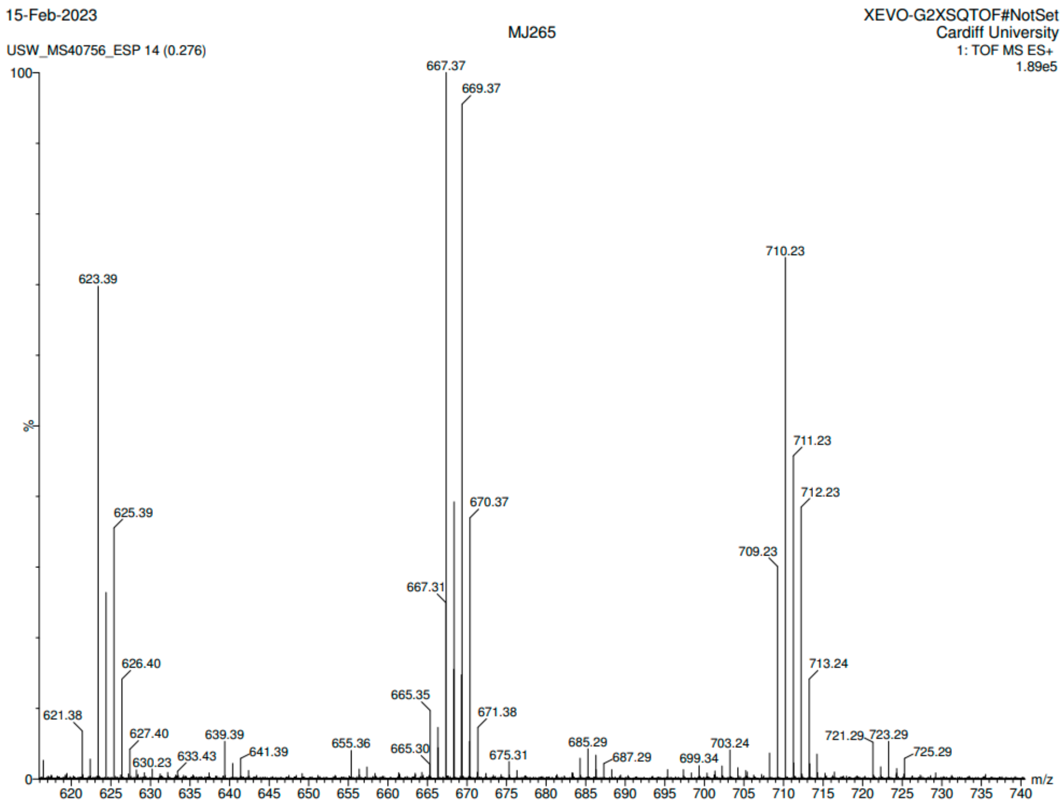

Fig S5.5: Mass spectrometry data for **[Cu{κ^3-*N,N,H*-^{Ph}Bai}(PCy$_3$)]**

B6 Spectra for [Cu{κ^3-*N,N,H*-^{Naphth}Bai}(PCy$_3$)] (6)

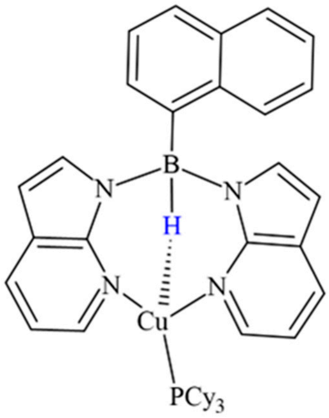

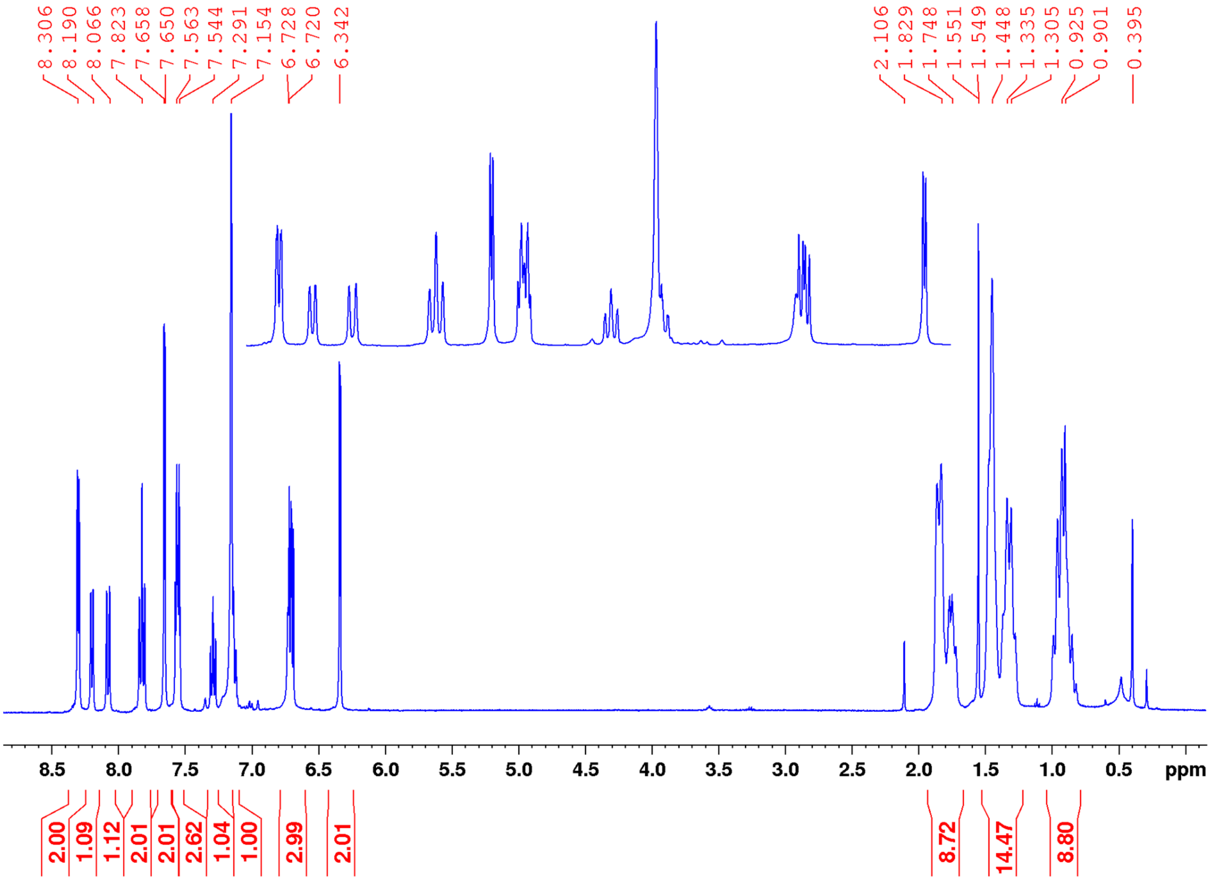

Fig S6.1: ^{1}H{^{11}B} NMR spectrum of the complex **[Cu{κ^3-*N,N,H*-^{Naphth}Bai}(PCy$_3$)]** C_6D_6 at 298 K.

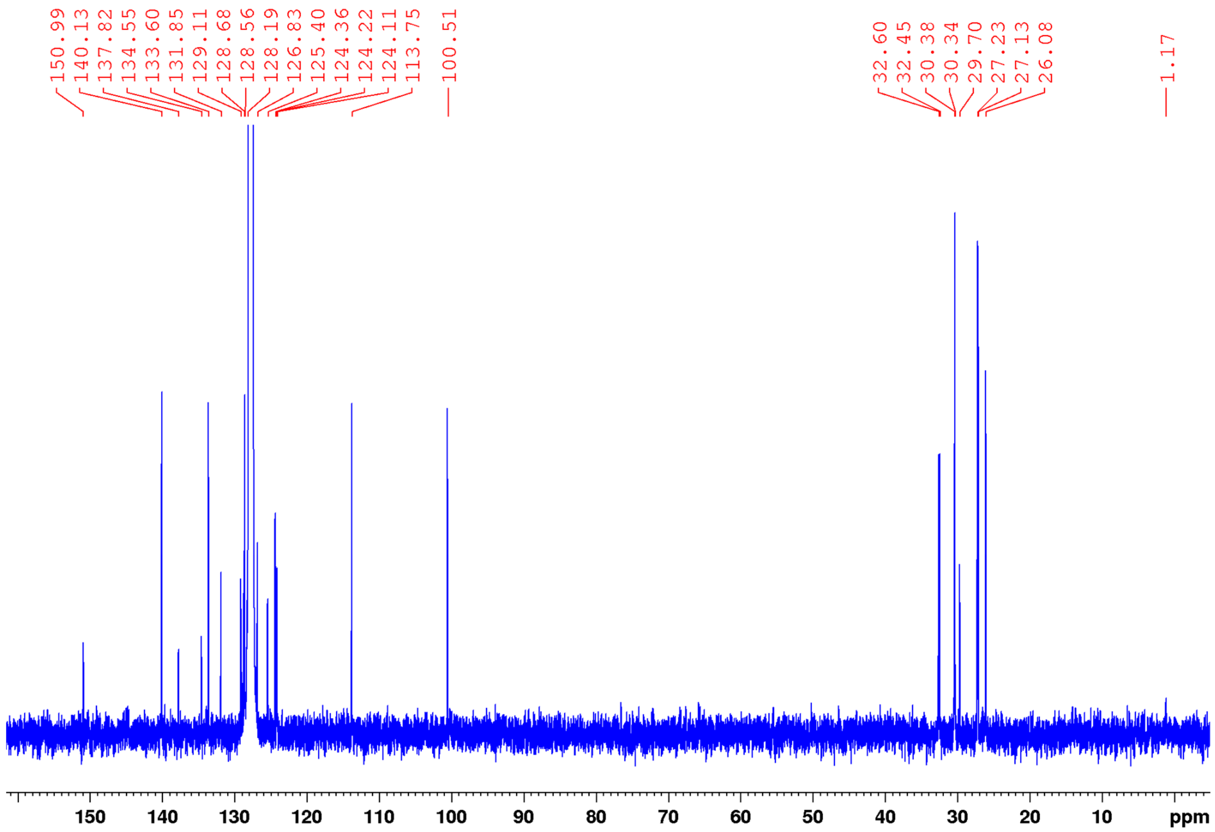

Fig S6.2: ^{13}C NMR spectrum of the complex **[Cu{κ^3-*N*,*N*,*H*-^{Naphth}Bai}(PCy$_3$)]** C_6D_6 at 298 K.

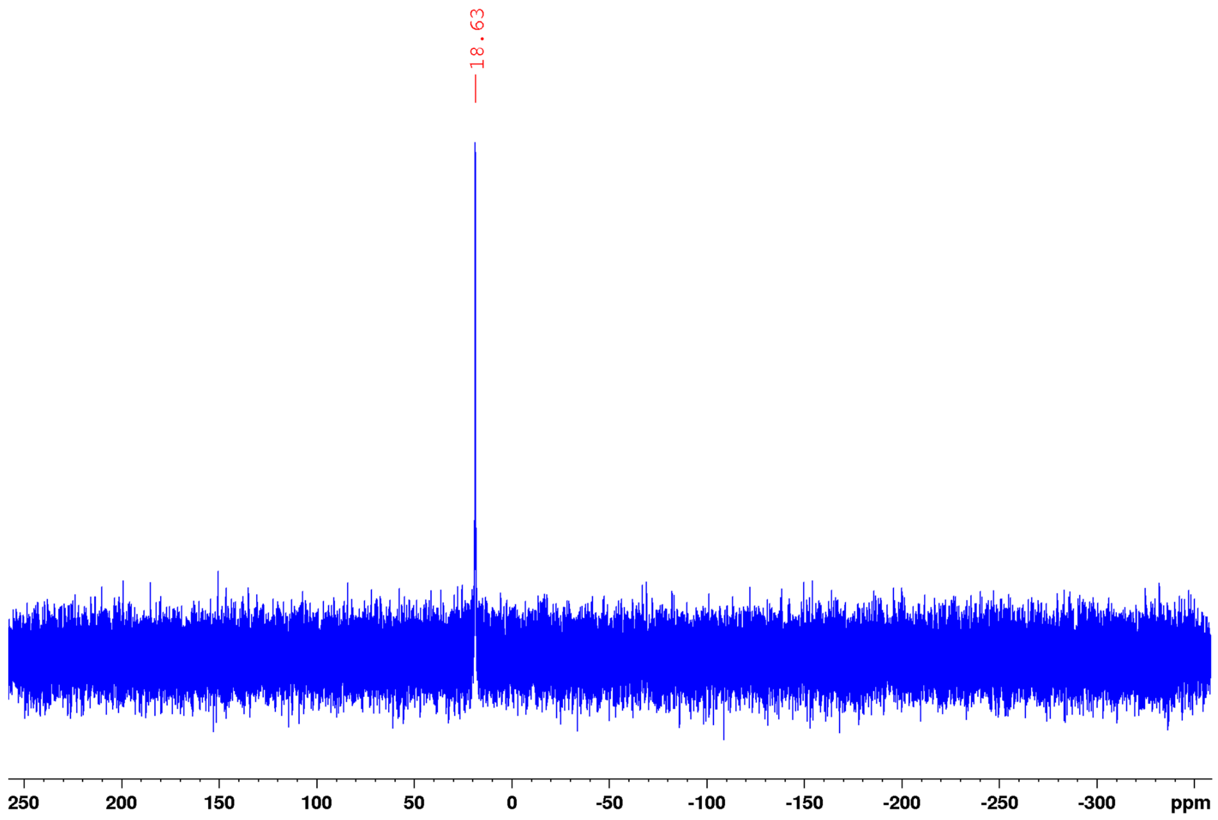

Fig S6.3: ^{31}P{^{1}H} NMR spectrum of the complex **[Cu{κ^3-*N*,*N*,*H*-^{Naphth}Bai}(PCy$_3$)]** C_6D_6 at 298 K.

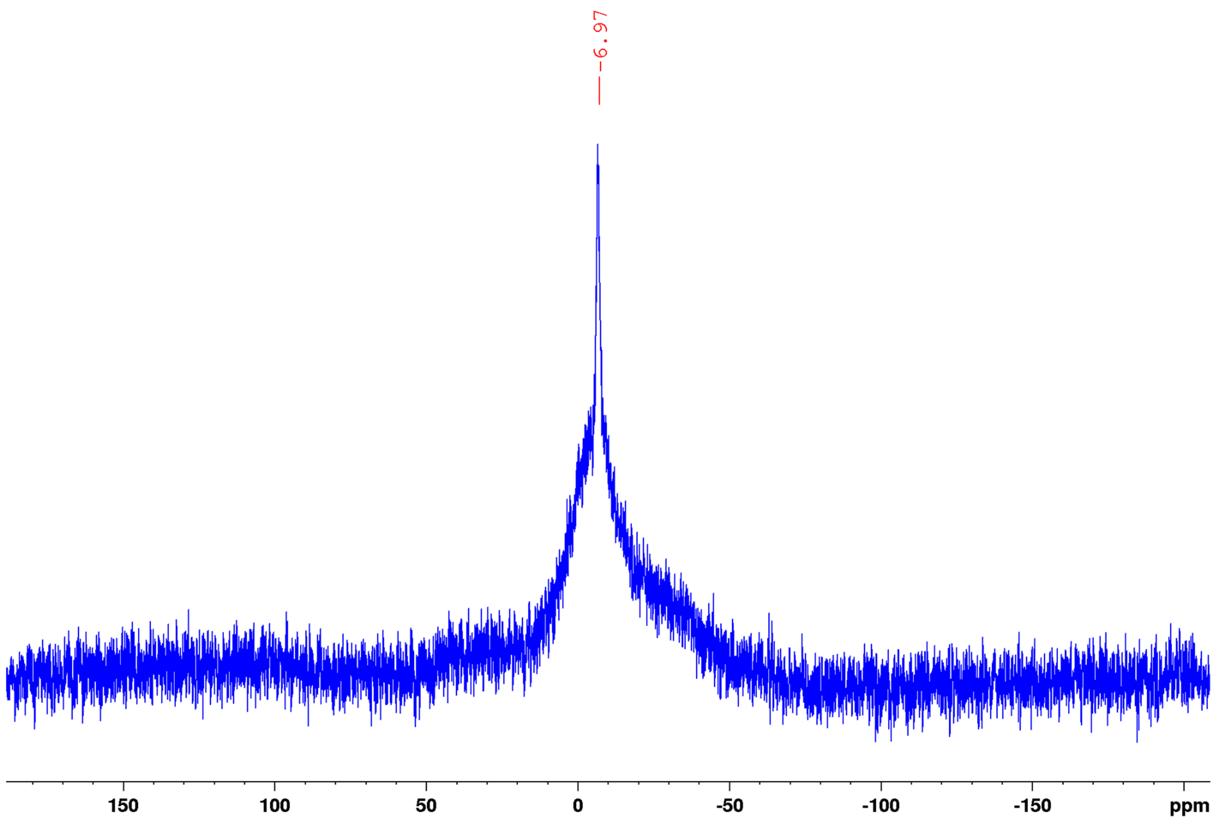

Fig S6.4: $^{11}B\{^{1}H\}$ NMR spectrum of the complex **[Cu{κ³-*N,N,H*-^{Naphth}Bai}(PCy₃)]** C_6D_6 at 298 K.

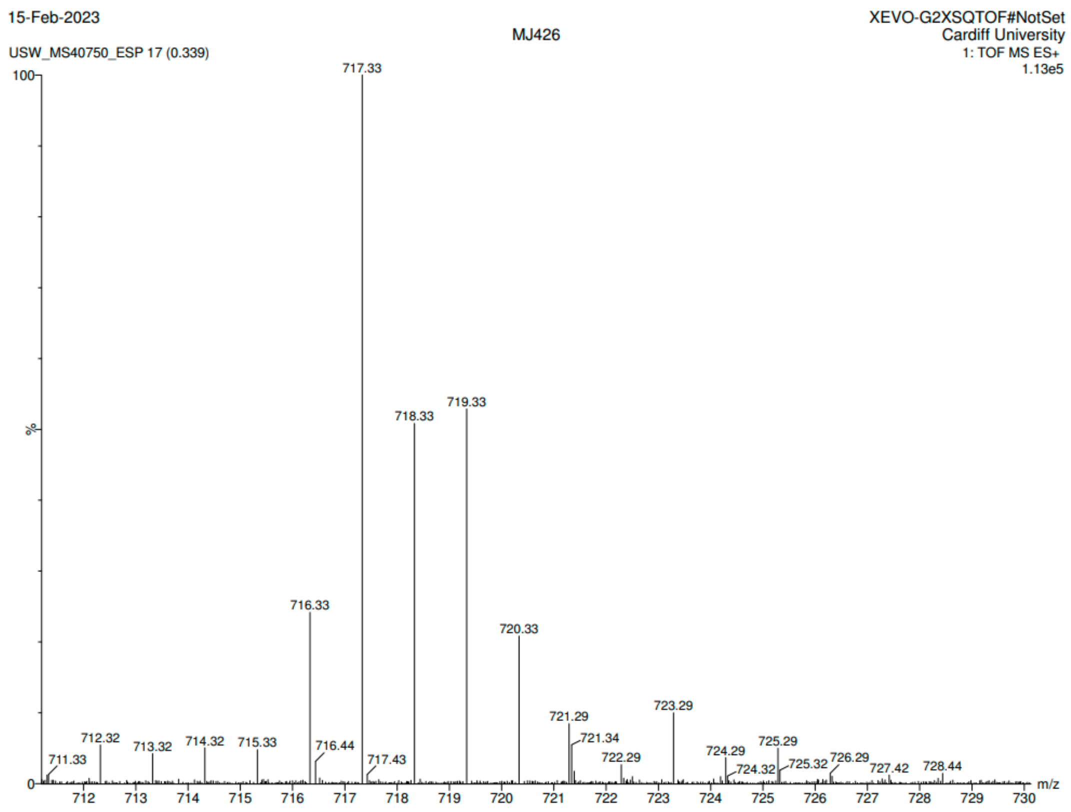

Fig S6.5: Mass spectrometry data for **[Cu{κ³-*N,N,H*-^{Naphth}Bai}(PCy_3)]**

B7 Spectra for [Cu{κ^3-*N,N,H*-^{Me}Bai}$_2$] (7)

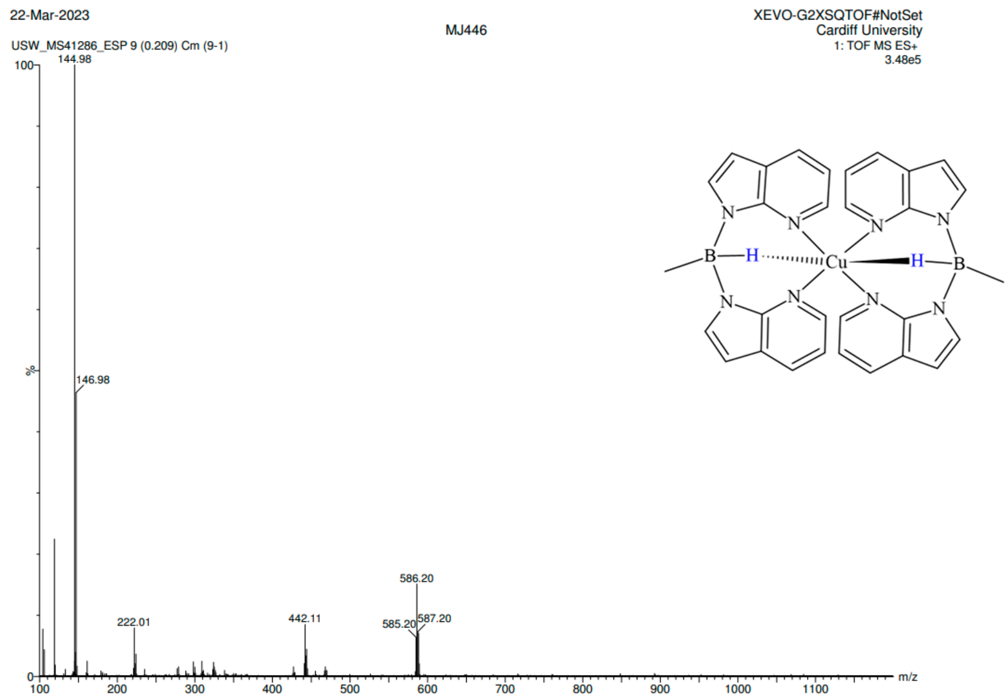

Fig S7.1: Mass spectrometry data for **[Cu{κ^3-*N,N,H*-^{Me}Bai}$_2$]**

B8 Spectra for [Cu{κ^3-*N,N,H*-^{Ph}Bai}$_2$] (8)

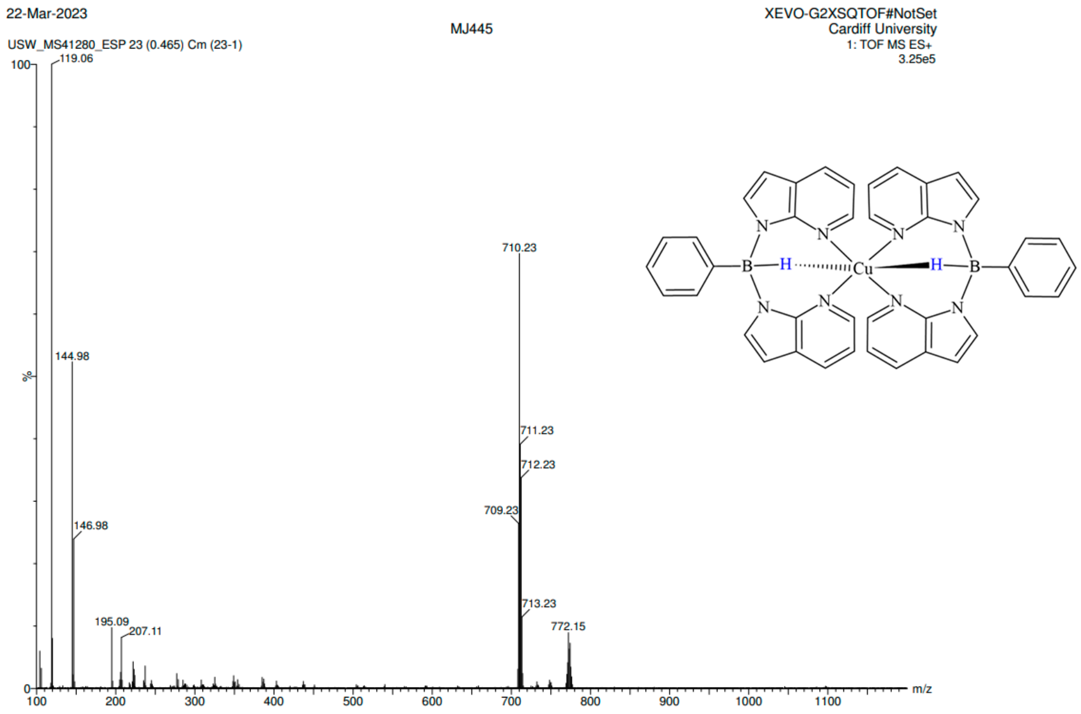

Fig S8.1: Mass spectrometry data for **[Cu{κ^3-*N,N,H*-^{Ph}Bai}$_2$]**

B9 Spectra for [Cu{κ^3-*N,N,H*-^{Naphth}Bai}$_2$] (9)

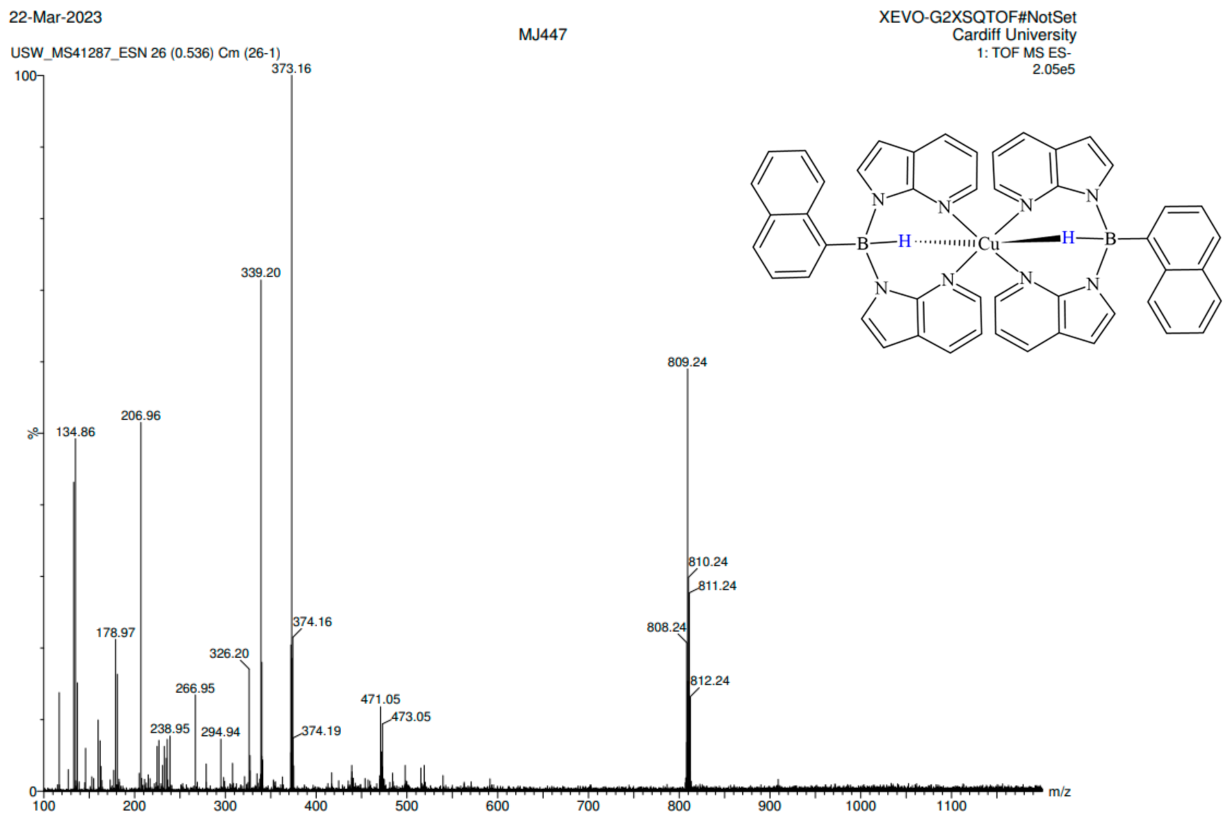

Fig S9.1: Mass spectrometry data for **[Cu{κ^3-*N,N,H*-^{Naphth}Bai}$_2$]**
